# LSD1-Based Reversible Inhibitors Virtual Screening and Binding Mechanism Computational Study

**DOI:** 10.3390/molecules28145315

**Published:** 2023-07-10

**Authors:** Zhili Yin, Shaohui Liu, Xiaoyue Yang, Mengguo Chen, Jiangfeng Du, Hongmin Liu, Longhua Yang

**Affiliations:** 1 School of Pharmaceutical Sciences, Zhengzhou University, Zhengzhou 450001, China; 202022342015572@gs.zzu.edu.cn (Z.Y.); lshaohui12138@163.com (S.L.); yxyang0206@163.com (X.Y.); m15239422053@163.com (M.C.); liuhm@zzu.edu.cn (H.L.); 2 School of Life Sciences, Zhengzhou University, Zhengzhou 450001, China; jiangfengdu@zzu.edu.cn; 3 Key Laboratory of Advanced Drug Preparation Technologies, Ministry of Education, Zhengzhou University, Zhengzhou 450001, China; 4 State Key Laboratory of Esophageal Cancer Prevention & Treatment, Zhengzhou University, Zhengzhou 450001, China; 5 Key Laboratory of Henan Province for Drug Quality and Evaluation, Zhengzhou University, Zhengzhou 450001, China; 6 Institute of Drug Discovery and Development, Zhengzhou University, Zhengzhou 450001, China

**Keywords:** virtual screening, pharmacophore model, molecular docking, LSD1 inhibitor, molecular dynamics simulations, binding free energy calculations

## Abstract

As one of the crucial targets of epigenetics, histone lysine-specific demethylase 1 (LSD1) is significant in the occurrence and development of various tumors. Although several irreversible covalent LSD1 inhibitors have entered clinical trials, the large size and polarity of the FAD-binding pocket and undesired toxicity have focused interest on developing reversible LSD1 inhibitors. In this study, targeting the substrate-binding pocket of LSD1, structure-based and ligand-based virtual screenings were adopted to expand the potential novel structures with molecular docking and pharmacophore model strategies, respectively. Through drug-likeness evaluation, ADMET screening, molecular dynamics simulations, and binding free energy screening, we screened out one and four hit compounds from the databases of 2,029,554 compounds, respectively. Generally, these hit compounds can be divided into two categories, amide (**Lig2** and **Comp2**) and 1,2,4-triazolo-4,3-α-quinazoline (**Comp3, Comp4, Comp7**). Among them, **Comp4** exhibits the strongest binding affinity. Finally, the binding mechanisms of the hit compounds were further calculated in detail by the residue free energy decomposition. It was found that van der Waals interactions contribute most to the binding, and **FAD** is also helpful in stabilizing the binding and avoiding off-target effects. We believe this work not only provides a solid theoretical foundation for the design of LSD1 substrate reversible inhibitors, but also expands the diversity of parent nucleus, offering new insights for synthetic chemists.

## 1. Introduction

**Figure 1:**
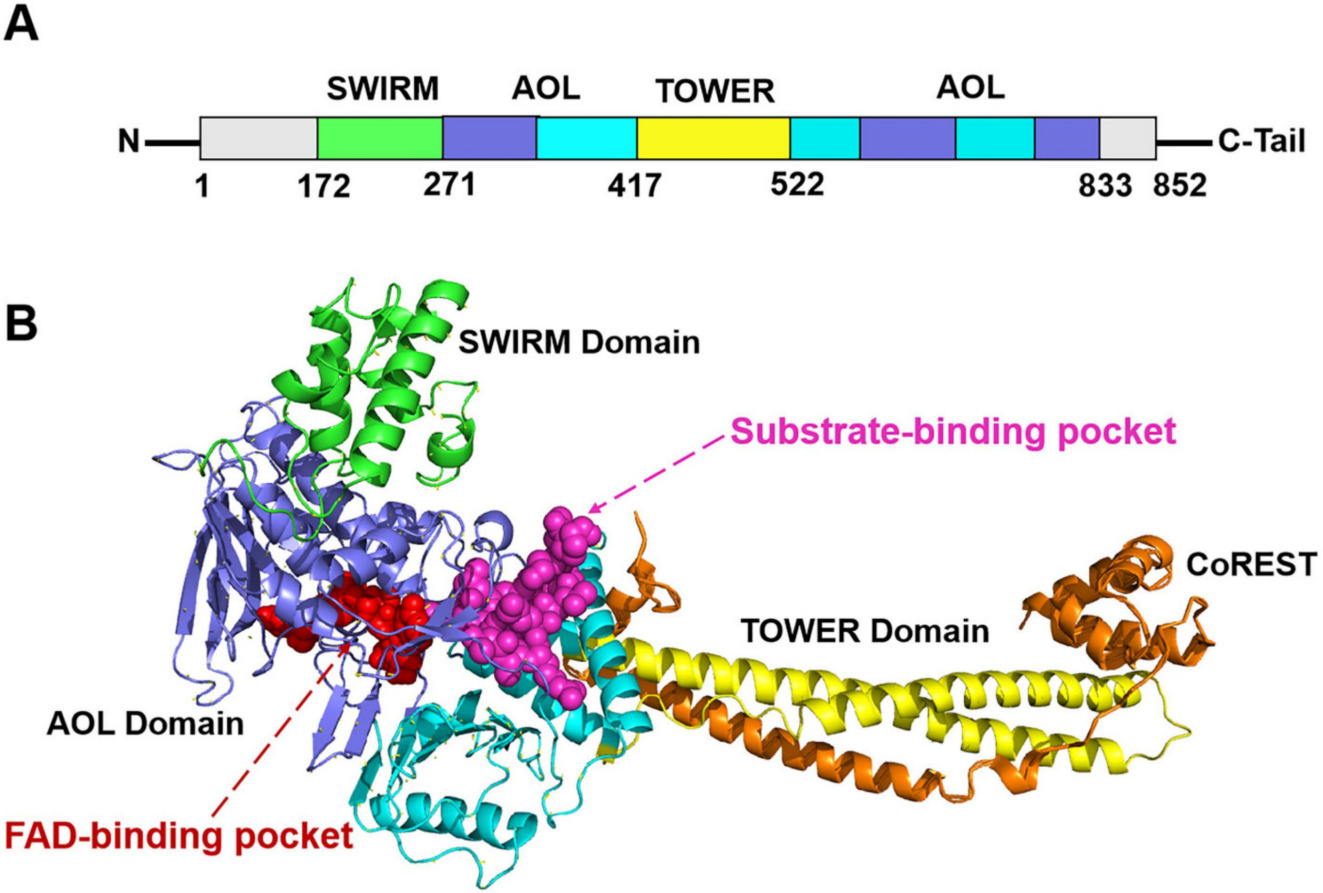
The (**A**) primary sequence and (**B**) tertiary structure schematic diagrams of LSD1 [9]. The FAD-binding pocket and substrate-binding pocket are colored in red and magenta, respectively.

Histone lysine-specific demethylase 1 (LSD1) is a highly conserved flavin adenine dinucleotide (**FAD**) dependent oxidative enzyme, the ability of expression of which has been discovered in a variety of tumor cells, including neuroblastoma, breast cancer, lung cancer, gastric cancer, acute myeloid leukemia, and so on [1,2,3,4,5]. LSD1 regulates tumor growth and metastasis through its demethylation function, which is determined by the molecules it binds. LSD1/REST or LSD1/CO-REST complexes are recruited to the corresponding promoter sequence and bind to it, removing the methyl group on the target gene through “epigenetic silencing” [4,5]. Structurally, the full-length LSD1 is comprised of 852 amino acids with three key structure domains: an N-terminal SWIRM domain (small alpha-helical domain, residues 172–271), a central protruding TOWER domain (residues 417–522), and C-terminal AOL (amine oxidase-like domain, residues 271–417 and 522–833) [6] (Figure 1). The SWIRM domain contributes to the steadiness of the protein and could participate in the LSD1-mediated protein-protein interactions that are imperative for regulating epigenetic modifications [7]. The TOWER domain is significant for maintaining the AOL domain spatial configuration, and could affect the interactions with other proteins, such as COREST [8]. The AOL domain consists of an FAD-binding domain and a substrate-binding domain, with two active binding centers in between at the crossover point. These two active binding sites form the catalytic activity center jointly.

**Figure 2:**
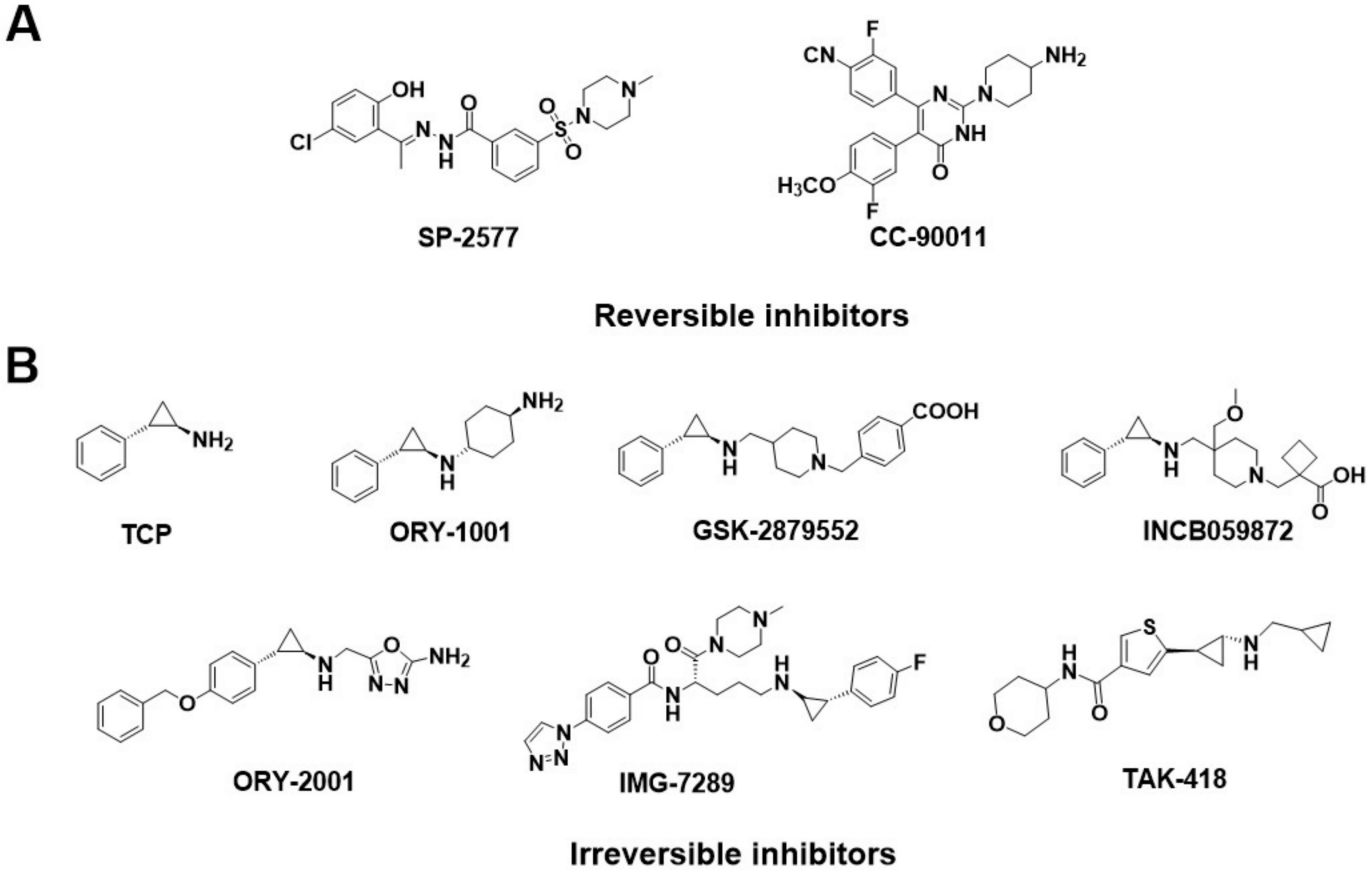
Structure of (**A**) reversible LSD1 inhibitors and (**B**) TCP-based irreversible LSD1 inhibitors in clinical trials.

Generally, the reported LSD1 inhibitors [10] can be divided into two categories: irreversible and reversible ones. Recently, almost all the irreversible LSD1 inhibitors already undergoing clinical trials are effective on the FAD catalytic pocket, most of which are tranylcypromine-based compounds, such as **TCP**, **ORY-1001** [11], **GSK-2879552** [12,13], **IMG-7289**, **INCB059872**, **ORY-2001**, and **TAK-418** (Figure 2B). These inhibitors bond with FAD covalently and thus always produce on-target toxicity [6,14,15,16]. Distinguishingly, reversible LSD1 inhibitors have been found to target either the FAD catalytic pocket or the substrate-binding pocket. For the FAD catalytic pocket, most reversible inhibitors are FAD-competitive inhibitors, such as 3-(piperidin-4-ylmethoxy)-pyridine derivatives [17] or 5-cyano-6-phenyl pyrimidine analogs [18], which have achieved excellent potency in biochemical and cellular assays. Unfortunately, these compounds exerted toxic effects as well [17,19,20]. Apart from that, the large size and polarity of the FAD-binding pocket were the other significant limitations of the FAD-competitive inhibitors. As a result, more interest has started to focus on reversible inhibitors that target substrate-binding pockets.

Several classes of LSD1 inhibitors, such as (4-cyanophenyl) glycine derivatives [19], 3,5-diamino-1,2,4-triazoles [21], 5-hydroxypyrazole derivatives [22], and 4-(pyrrolidine-3-yl) benzonitrile derivatives [23], have been discovered to be with conspicuous bioactivities targeting the substrate-binding pocket. Among them, two compounds, **SP-2577** [24,25] and **CC-90011** [26], have already entered the clinical stage (Figure 2A). Replacing the morpholine of compound **SP-2509** with N-methylpiperazine, Sorna et al. [25] obtained compound **SP-2577** (K_i_ = 31 nM, IC_50_ = 13 nM). This modification overcame the off-target effects of **SP-2509** and has already started the phase I clinical trials of the relapsed or refractory ES (NCT03600649) and advanced solid tumors (NCT03895684) [24]. In 2020, employing the high-throughput screening (HTS) technology and SAR method, Kanouni et al. [26] identified compound 5-chloro-4-(4-isocyanophenyl)-N-(pyrrolidin-3-ylmethyl)pyrimidin-2-amine. Through a set of structural replacement of the branch chain, the most active compound **CC-90011** (IC_50_ = 0.25 nM) was obtained. Additionally, the substrate-binding pocket can regulate the rotation of the amine oxidase domain concerning the TOWER domain, thereby affecting the overall receptor flexibility [27]. Thus, targeting the substrate-binding pocket would be a vital strategy for the design of corresponding reversible LSD1 inhibitors.

As one of the economical and effective methods for discovering small molecule lead compounds, the most popular virtual screening methods, structure-based and ligand-based virtual screenings (SBVS and LBVS), has been widely applied to target specific proteins. SBVS primarily utilizes the strategy of molecular docking, while LBVS includes QSAR, the pharmacophore model, and other strategies. For LSD1, Zhou et al. [28] performed a pharmacophore-based virtual screening of a library containing approximately 170,000 compounds and identified nine novel FAD-competitive LSD1 inhibitors in 2015. Compound **XZ09** exhibited the best inhibitory activity, with an IC_50_ of 2.41 uM. However, the virtual screening method has not been applied to the substrate-binding pocket, which can compensate for the scarcity of reversible inhibitors. 

In this study, targeting the substrate-binding pocket, LSD1 reversible inhibitors with strong specificity and high affinity were screened by structure-based and ligand-based virtual screenings, utilizing molecular docking and pharmacophore model strategies, respectively. Then, the corresponding binding mechanisms of hit compounds were explored through MD simulations and binding free energy calculations. We believe that this work is valuable for expanding the diversity of parent nucleus of LSD1 substrate reversible inhibitors, and also provides definite theoretical guidance for its further optimization for synthetic chemists.

## 2. Results and Discussion

### 2.1. Structure-Based Virtual Screening (SBVS)

#### 2.1.1. Drug-Likeness and ADMET Screening

**Scheme 1:**
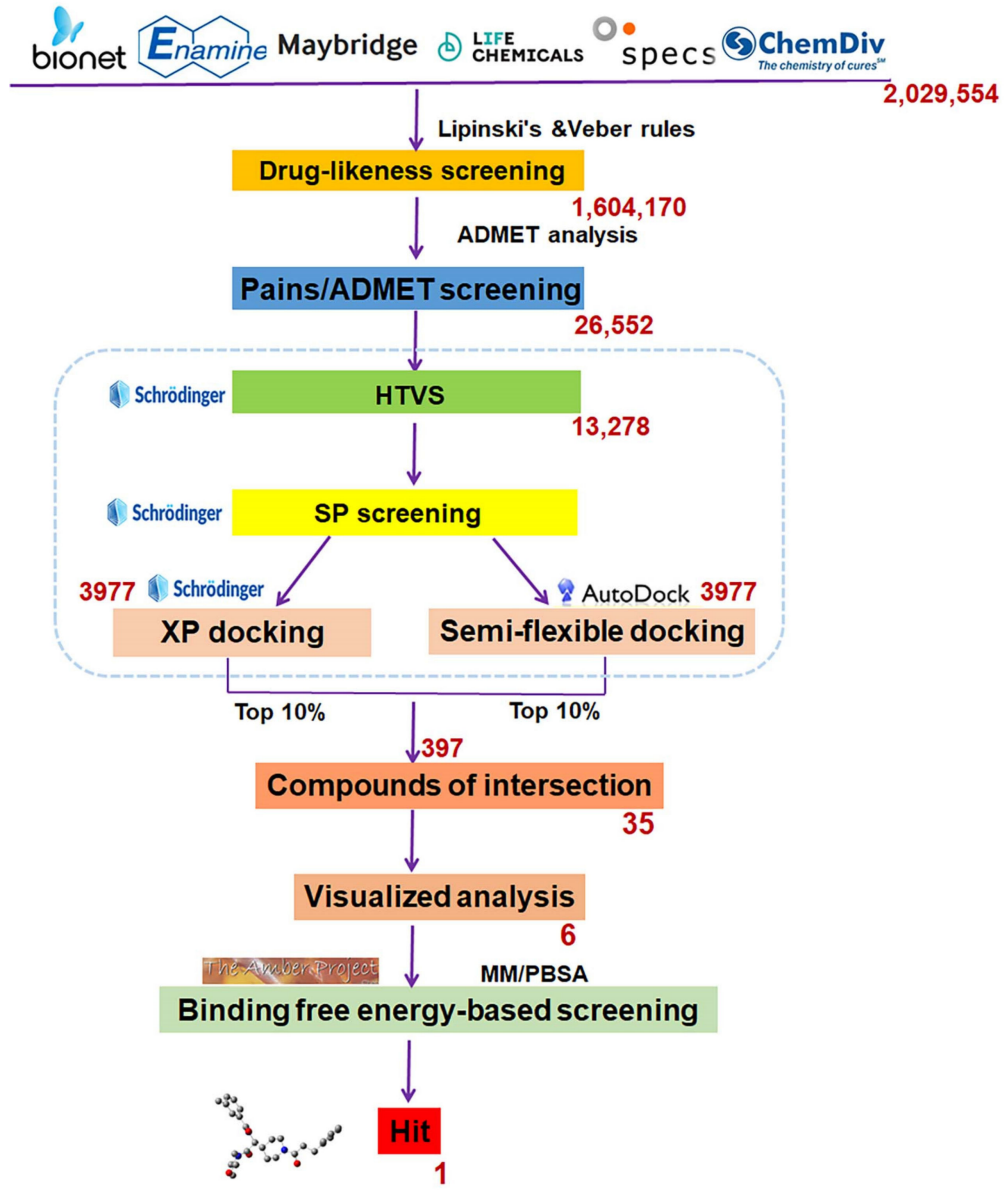
The flowchart of structure-based virtual screening targeted the substrate-binding pocket of LSD1. The red number represents how many compounds are remained after each round of screening.

Scheme 1 showed the complete SBVS flow diagram. Drug-likeness screening was performed to expedite the narrowing of the range first, and 1,604,170 compounds were screened out following Lipinski’s and Veber’s rules. Then, 26,556 compounds with favorable ADMET properties were retained for subsequent molecular docking after ADMET screening.

#### 2.1.2. Molecular Docking Studies

##### Selection of Docking Methods

In order to verify the reliability of the docking algorithm, three docking programs (Molecular Operating Environment (MOE2020), Schrödinger, and Autodock Vina) were compared and evaluated using the positive control **CC-90011** (PDB ID: 6W4K) [26]. The evaluation criterion was to check whether a docking program can effectively reproduce the binding modes of ligands in crystal structures of LSD1-ligand complexes. The corresponding root mean squared deviation (RMSD) values after docking are 1.449 Å (MOE), 0.45 Å (Schrödinger), and 0.003 Å (Autodock Vina), all falling within the error range (2 Å) for SBVS. Obviously, AutoDock Vina and Schrödinger docking programs were more favorable than MOE2020 and were chosen to perform the following molecular docking screening.

##### Docking Screening

Three graded dockings were conducted following the previously described docking workflow and criteria for Molecular docking study in SBVS. Initially, a rough HTVS was performed, followed by a more precise SP docking screening, obtaining 3977 screened compounds. Subsequently, based on the score ranking, the top 10% of compounds obtained from XP docking in Schrödinger and the semi-flexible docking in Autodock Vina were intersected, retaining 35 compounds of intersection. Finally, following the standard that at least one hydrogen bond interaction existed between the compounds and residue Asp555 or Lys661, only six compounds (Table 1) were retained through visualized analysis. The drug-likeness and ADMET screening results are presented in Table S3.

#### 2.1.3. Screening Based on Binding Free Energy Calculations

Based on the LSD1-ligand docking conformations with the highest score among the six screened compounds, 175 ns MD simulations were performed, and the corresponding binding free energies were calculated using the last 50 ns of equilibrated trajectories. Figure S1 shows the RMSD (Cα atom) and root mean square fluctuations (RMSF) curves of these six complex systems, as well as the **sole-LSD1** and **LSD1_CC-90011** positive control systems. Comparing their average RMSD values, it was observed that except for **Lig1** and **Lig5**, the interactions of the other four complexes with LSD1 slightly increased the system stabilities compared to the **sole-LSD1** (3.45 Å) system (Table S4). The RMSF results indicated that two amino acid sequences (450–493 and 783–793) exhibit significant fluctuations. Structurally, since these two sequences belong to the inherently flexible loop region and are far from the targeted binding pocket, the fluctuations would not affect the binding mode.

With the MM/PBSA method, the binding free energies of six complexes and the **LSD1_CC-90011** positive control system were computed based on the last 50 ns trajectories. The binding free energy of **CC-90011** with LSD1 was also calculated as a reference, and the results were presented in Table 2. It is generally believed that the lower the ΔG_bind_ value, the more stable the complex. As shown in Table 2, the ΔG_bind_ of **Lig2** is −63.13 kcal/mol, more negative than **CC-90011** (ΔG_bind_ = −44.42 kcal/mol), which suggests that it may be a potential inhibitor of LSD1. Furthermore, the favorable contributions to ΔG_bind_ are the same, mainly coming from ΔE_ele_, ΔE_vdw_, and ΔG_nonp_. Among these three energies, ΔE_vdw_ consistently has the most negative value, indicating its significant role as the driving force in maintaining the stability between protein and ligands. In contrast with ΔE_vdw_, ΔG_PB_, with the largest positive value, makes an unfavorable contribution to the binding free energy.

#### 2.1.4. Binding Mode Analysis

**Figure 3:**
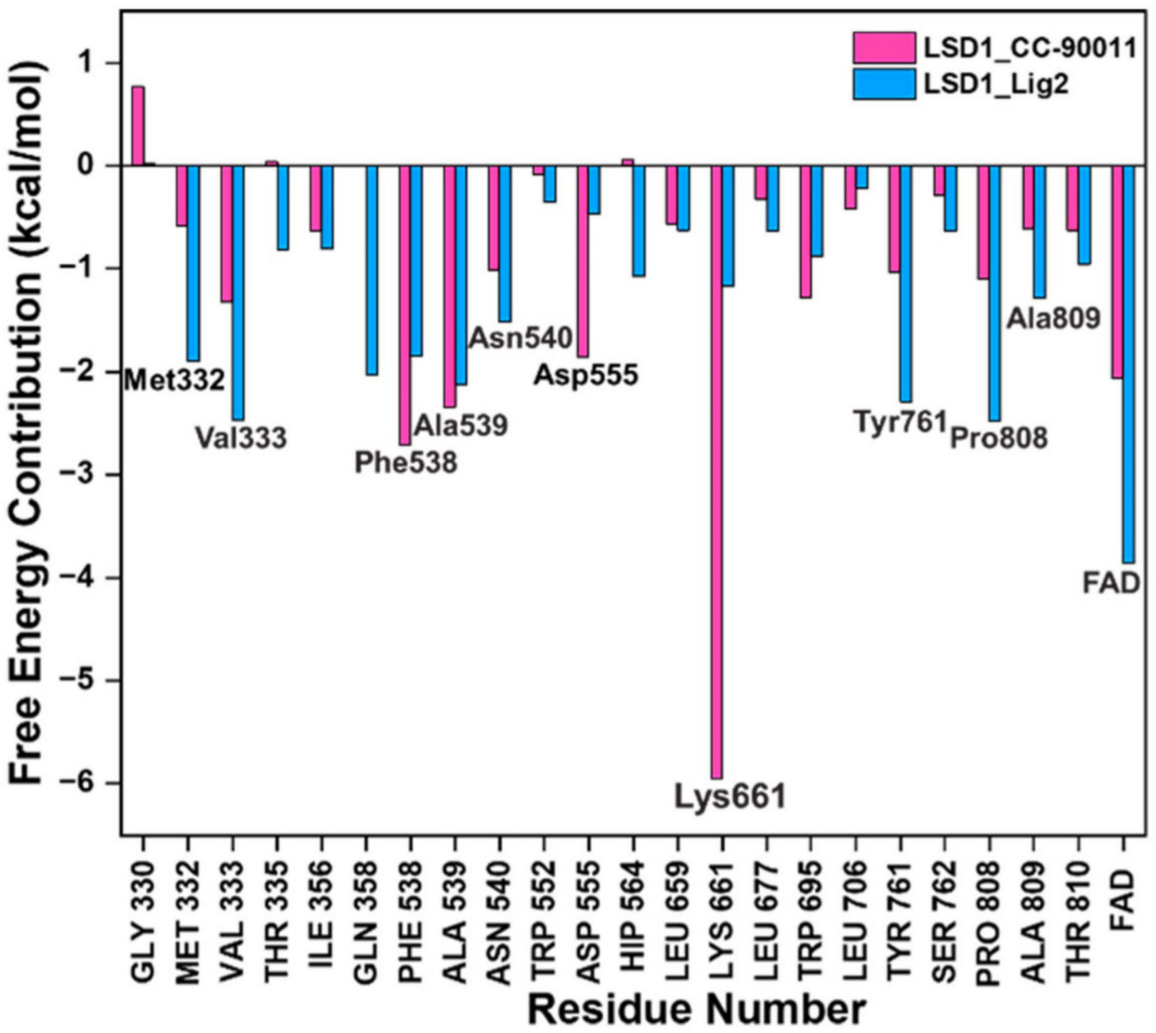
Residues free energy decomposition calculations for the binding pocket composed in **LSD1_CC-90011** and **LSD1_Lig2** complexes.

To gain further insights into the binding mode of **Lig2** and the binding pocket of LSD1, taking **CC-90011** as the positive control, the corresponding residue free energy decomposition was calculated, and values more negative than −1 kcal/mol are listed in Figure 3. When comparing **LSD1_CC-90011** and **LSD1_Lig2** complex systems, there are seven common residues, including Lys661, Phe538, la539, Val333, Pro808, Tyr761, and Asn540. Among these residues, four are hydrophobic (Phe538, Ala539, Val333, and Tyr761), indicating that hydrophobic interactions are the primary interactions involved. This is also inconsistent with the binding free energy calculation results. The only difference is that in LSD1_CC-90011, Lys661 has a major favorable contribution, with a decomposition energy of −5.95 kcal/mol, much larger than that of **Lig2** (Table S5). This energy gap is offset by the larger hydrophobic interaction between **FAD** and **Lig2**, with a decomposition energy of −3.85 kcal/mol, which is greater than that of **CC-90011** (−2.06 kcal/mol) (Table S5). In this way, the **FAD** in the catalytic pocket is associated with the hit **Lig2** together, ensuring its stability in the adjacent substrate-binding pocket.

### 2.2. Ligand-Based Virtual Screening (LBVS)

#### 2.2.1. Pharmacophore Model Generation

**Figure 4:**
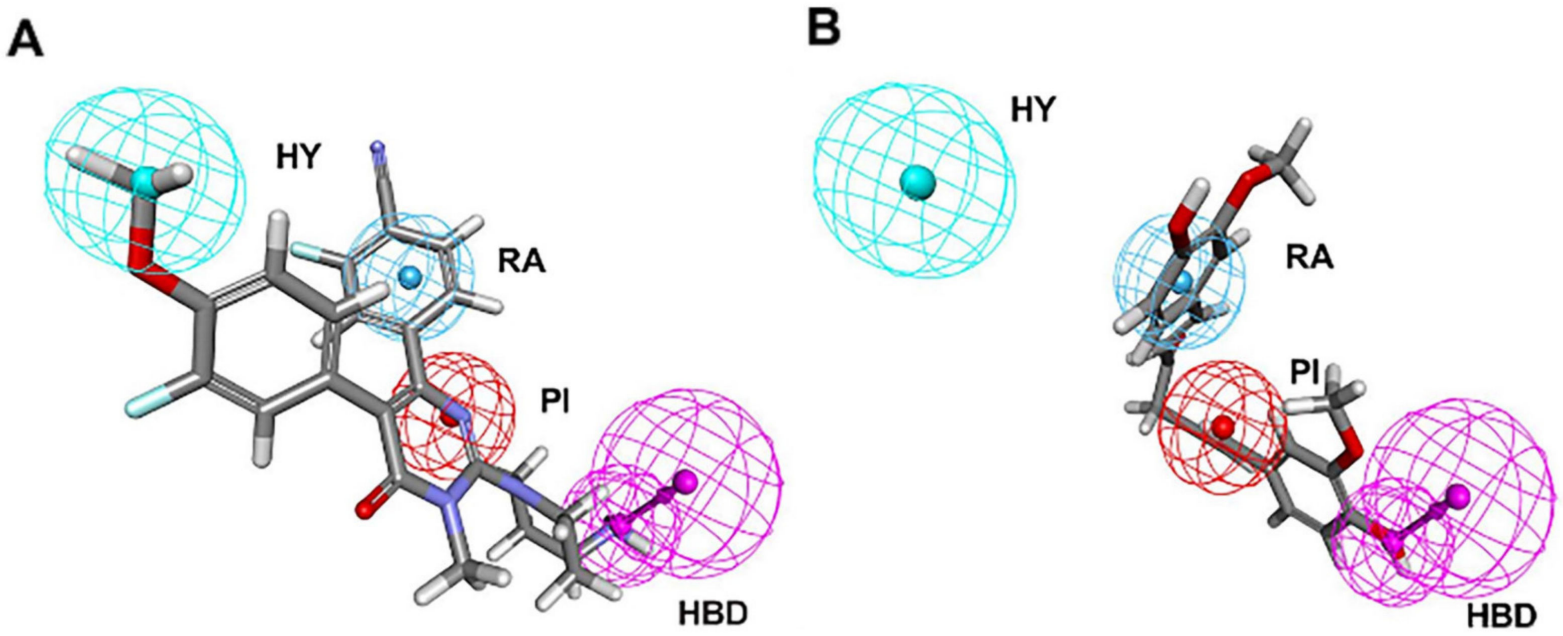
Pharmacophore mapping of (**A**) the most active compound **1** and (**B**) the lowest activity compound **21** on the best hypothesis model HypoGen1. HY, RA, PI, HBD are colored in cyan, blue, red and magenta, respectively.

As listed in Table 3, 10 HypoGen pharmacophore models were generated, aiming to use 3D alignments of molecules further to predict the activity of compounds quantitatively. According to Occam’s razor [29,30] principle, the best pharmacophore model should exhibit the highest correlation coefficient, lowest cost value, highest cost difference, and lowest RMS value. Consequently, HypoGen1 consists of HY, RA, PI, and HBD features, was statistically identified as the significant hypothesis, and was chosen to estimate the activities of 24 compounds in the test set (Figure 4). The calculated and experimental activities of all compounds in the training set are shown in Table 4.

**Figure 5:**
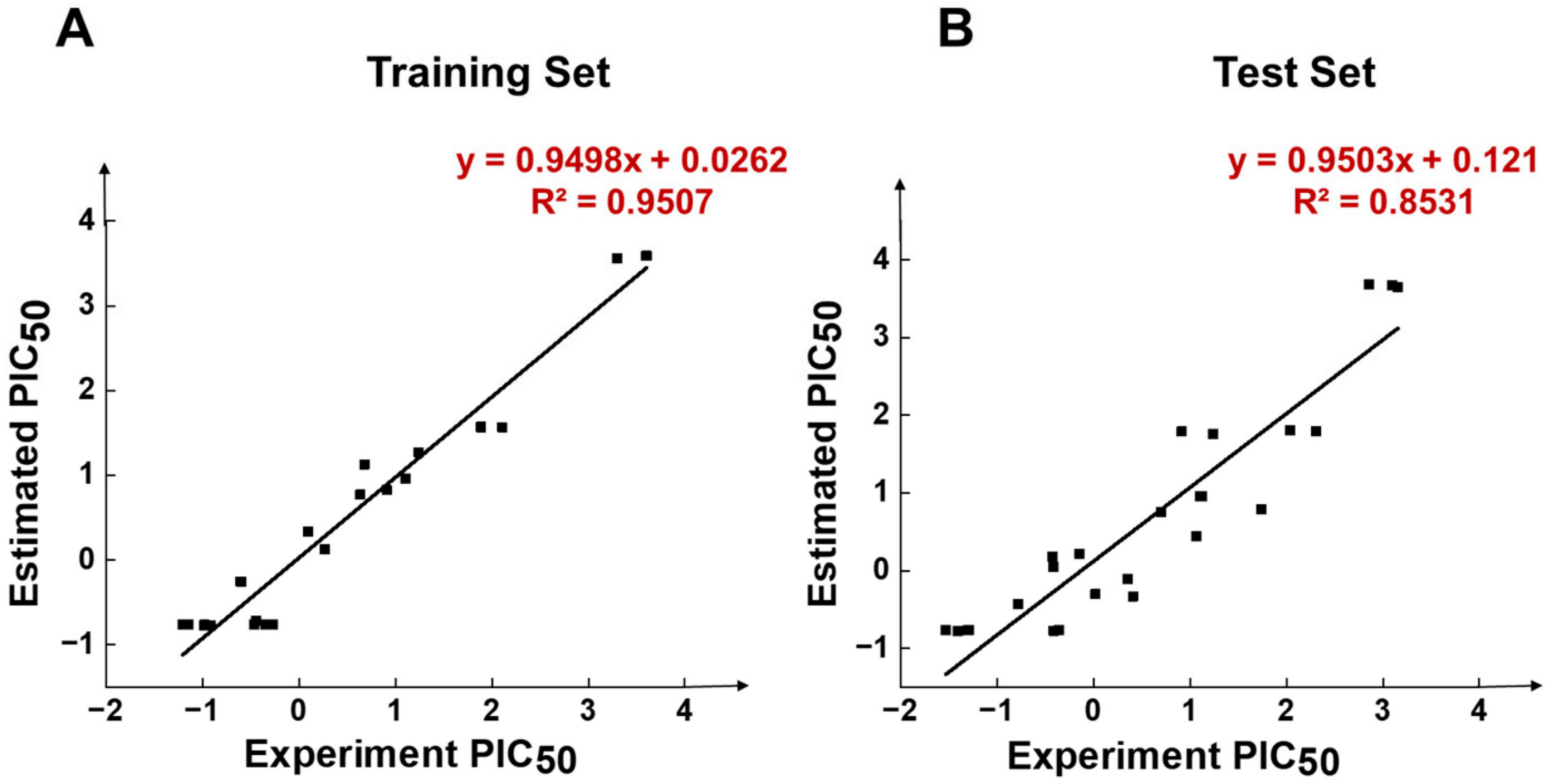
Scatter diagrams of experimental and predicted activity of compounds in HypoGen1 pharmacophore in (**A**) Training set, and (**B**) Test set.

In Figure 4A, Compound **1** was mapped onto PI, HBD, RA, and HY features of HypoGen1 with a fit value of 9.71, while for Compound **21** with the lowest activity values, the matching results are not as satisfactory, thus showcasing the competence of HypoGen1 in selecting the most active compounds upon subjecting it to screen the databases (Figure 4B). Figure 5A illustrates the scatter plot between the experimental and estimated activity values of the compounds in the training set, the correlation coefficient of which was 0.9507. It indicates that the generated pharmacophore model can accurately predict the activity values of the compounds in the training set.

#### 2.2.2. Pharmacophore Model Validation

##### Cost Analysis

The best hypothesis was selected through cost analysis. According to the results (Table 3), HypoGen1 has the lowest total cost (97.64), closest to the fixed cost (65.47), and far away from the null cost (685.76) in comparison to the other hypotheses. Moreover, HypoGen1 demonstrates the highest correlation value of 0.98 and the lowest RMS value of 1.68. The cost difference is 588.12, much greater than 60 bits difference, indicating a greater than 90% chance of representing a true correlation in the data set used [31]. The above data indicate that HypoGen1 has good predictive capability.

##### Test Set Validation

HypoGen1 was verified by the test set to check its ability to predict compound activity accurately. A test set consisting of 24 compounds was mapped on HypoGen1, and a scatter plot comparing their experimental and estimated activities was generated (Table S6). As shown in Figure 5B, all test set samples are clustered around y = x, and the correlation coefficient R^2^ calculated is 0.8531, further confirming the suitability of HypoGen1 for activity prediction.

##### Fischer’s Randomization Test Analysis

**Figure 6:**
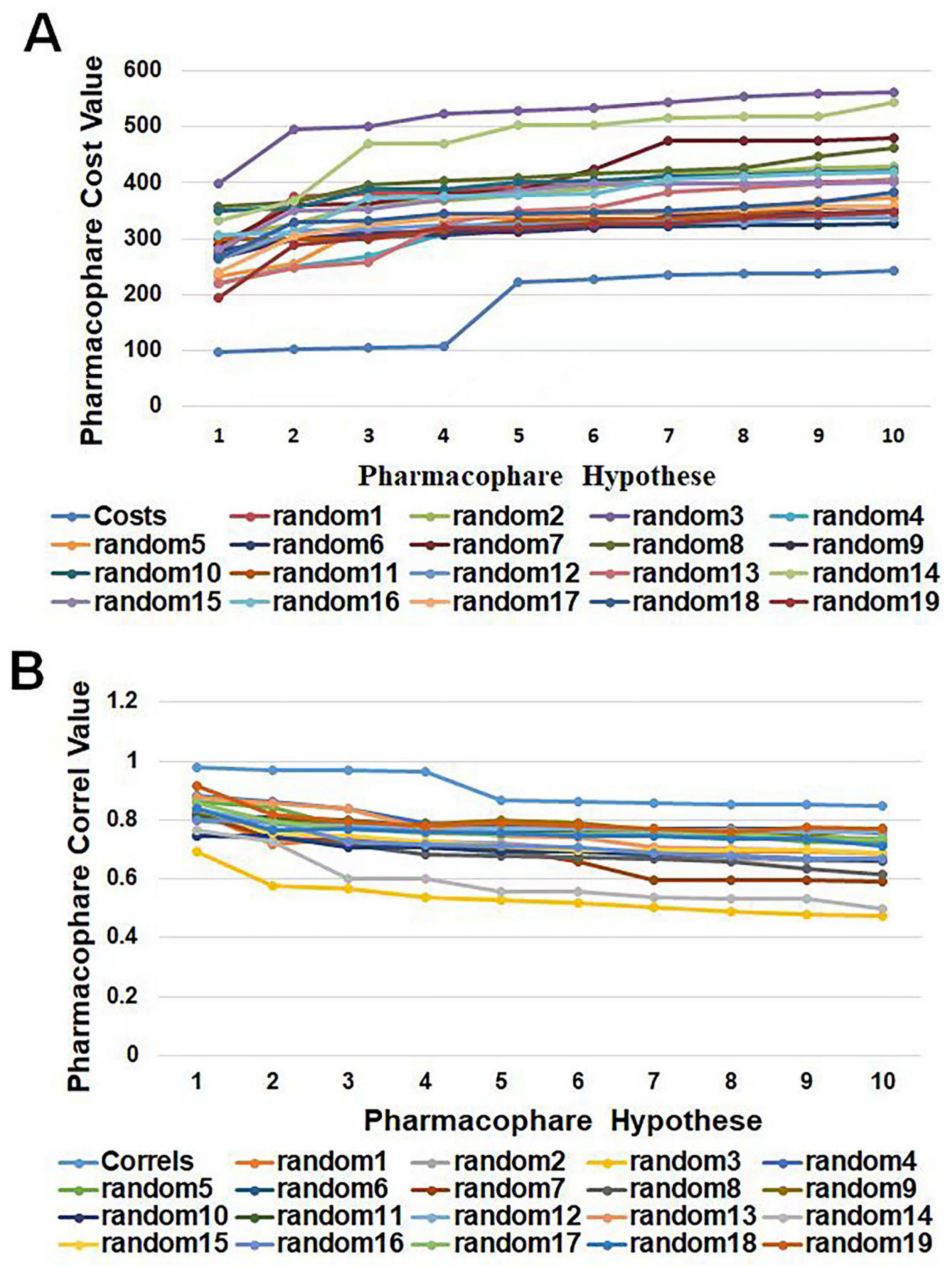
A graphical representation of the total cost values (**A**) and correl values (**B**) of HypoGen1 and ten hypotheses generated from 19 random spreadsheets during Fischer’s randomization run. A confidence level of 95% was used.

HypoGen1 was validated by Fischer’s randomization test to check the statistical significance, as well as to confirm whether the assumptions generated by the training set are reasonable. The experimental activity data of the compounds in the training set were randomly recombined to provide a “random” data set to create the pharmacophore model by using the same features and parameters as the initial pharmacophore model. Overall, 19 pharmacophore models were randomly generated, and the data line graph is shown in Figure 6. It can be observed that the cost value of HypoGen1 is lower than that of the randomly generated pharmacophore, and HypoGen1 has a higher correlation value compared to the 19 random hypotheses. These results indicate that this constructed pharmacophore model is superior and is not generated by chance at a 95% confidence level.

#### 2.2.3. Drug-Likeness Screening

**Scheme 2:**
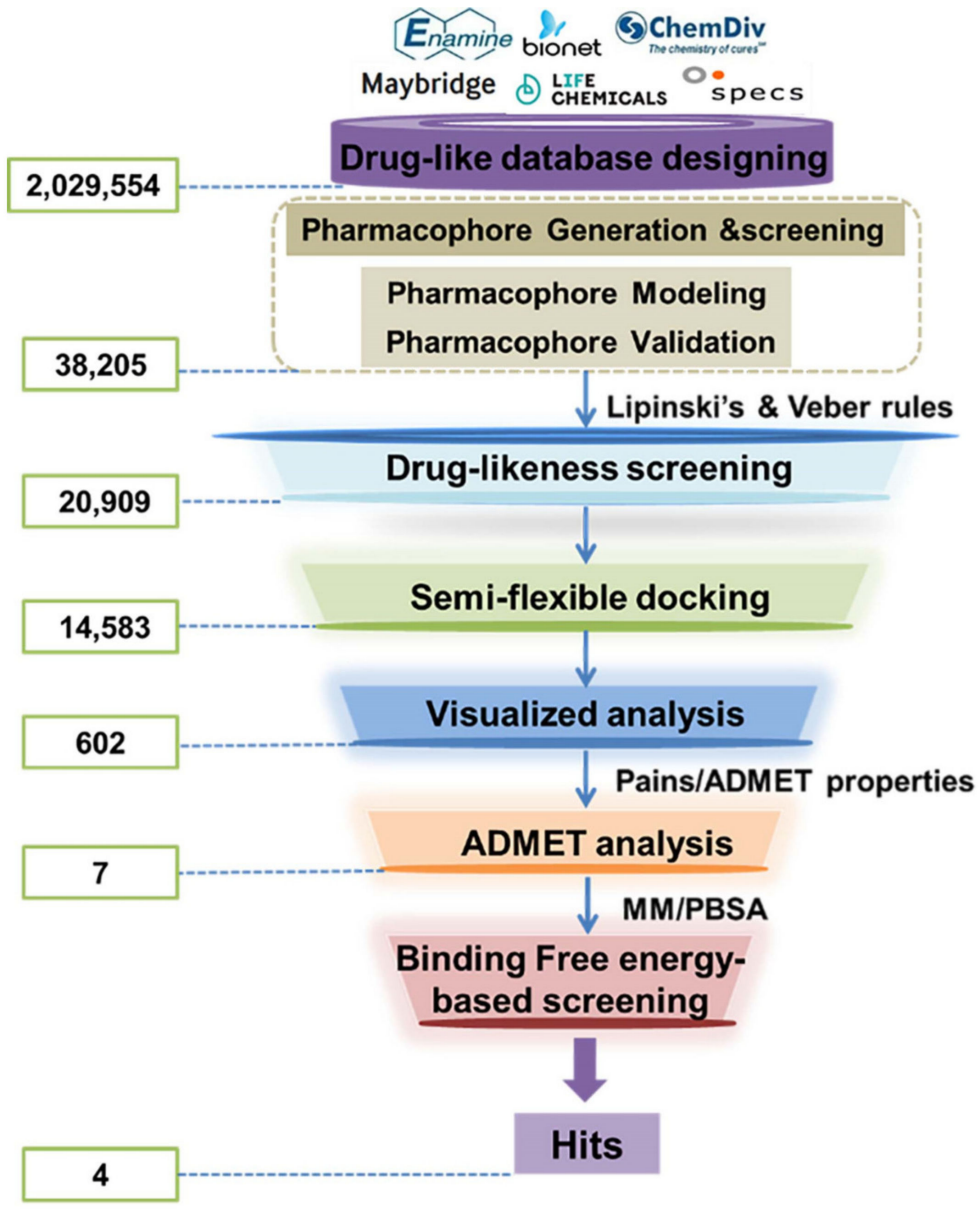
The flowchart of ligand-based virtual screening targeted the substrate-binding site of LSD1.

After evaluation, the verified HypoGen1 pharmacophore model was used for filtering LSD1 inhibitors from the databases (2,029,554 compounds). Upon applying the Ligand Pharmacophore Mapping module of DS2017R2, 38,205 compounds were retrieved. LBVS approach was performed through the drug-likeness screening, molecular docking, virtual analysis, and ADMET screening subsequently. The screening protocol is illustrated in Scheme 2. Following Lipinski’s and Veber’s rules, 20,909 compounds were selected for molecular docking studies.

#### 2.2.4. Screening Based on Docking, ADMET and Binding Free Energy

The top-scoring compounds (top 70%) were screened out after docking by AutoDock Vina. They were subsequently processed visualized analysis with the same standards as Selection of Docking Methods, finally obtaining 602 compounds. After ADMET screening, only seven compounds (Table 5) with favorable ADMET properties were retained for further analysis (Table S7).

Then, 200 ns MD simulations and binding free energy calculations of the complex systems composed of these seven selected compounds and LSD1 were performed to narrow down the region of interest. Compared their average RMSD values with that of **sole-LSD1** and **LSD1_CC-90011** positive control systems (Figure S2), it was observed that except for **Comp6**, the interactions of the other six complexes with LSD1 all slightly increase the system stabilities in comparison to the **sole-LSD1** (3.45 Å) system (Table S4). The calculated binding free energy results show that the ΔG_bin_d of **Comp2**, **Comp3**, **Comp4**, and **Comp7** (−48.51 kcal/mol, −51.06 kcal/mol, −54.26 kcal/mol, and −46.45 kcal/mol) are all more negative than that of **CC-90011** (−44.42 kcal/mol) (Table 6). This indicates that these compounds have greater binding affinities with LSD1, and thus can be considered as potential LSD1 inhibitors. Among them, **Comp4** has the highest binding free energy. Similarly, the favorable contributions to ΔG_bind_ are the van der Waals.

#### 2.2.5. Binding Modes Analysis

**Figure 7:**
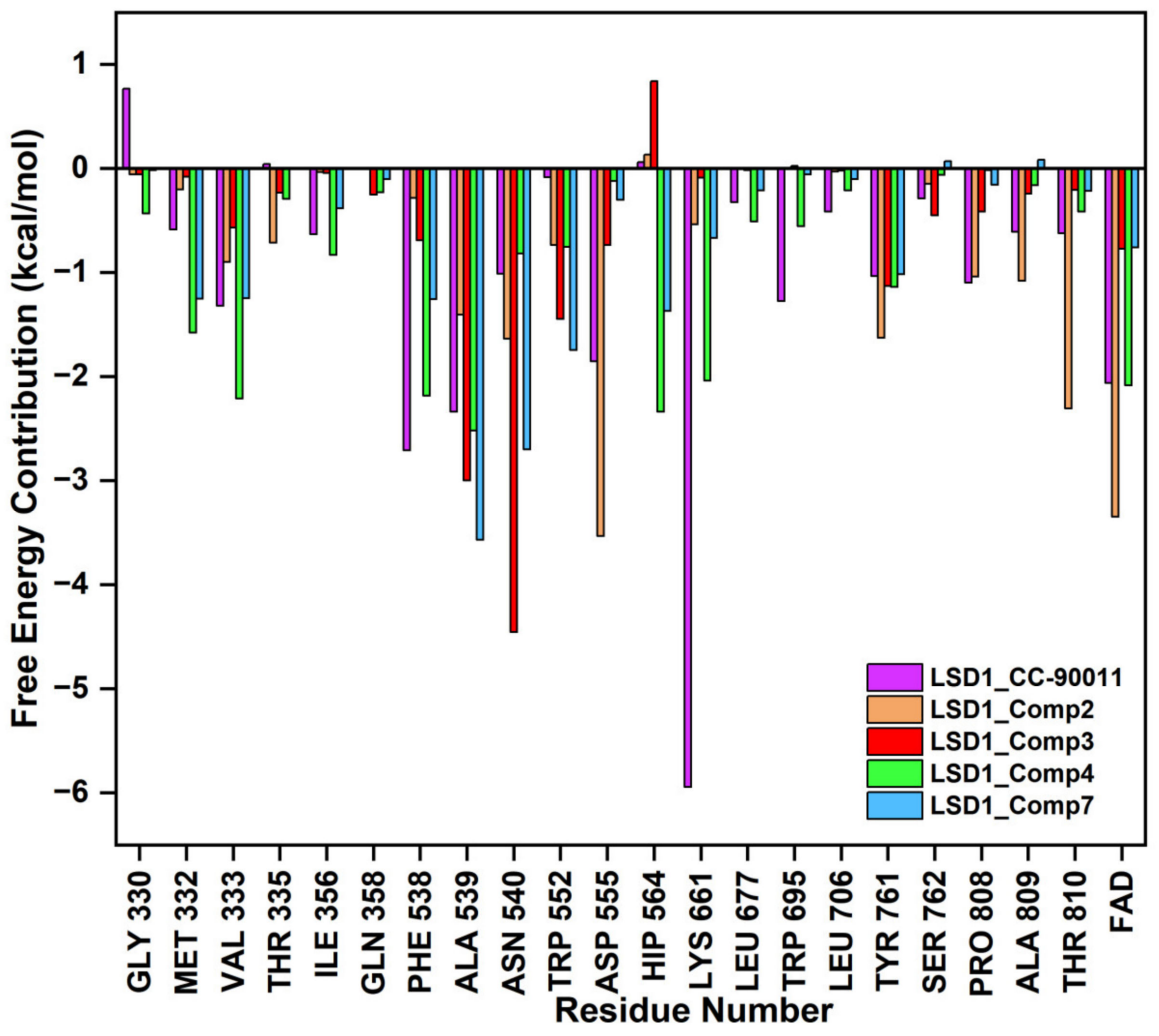
Residues free energy decomposition calculations for the binding pocket-composed in **LSD1_CC-90011**, **LSD1_Comp2**, **LSD1_Comp3**, **LSD1_Comp4**, and **LSD1_Comp7** complexes.

The binding modes of the filtered compounds **Comp2**, **Comp3**, **Comp4**, and **Comp7** with LSD1, as well as the positive control **CC-90011**, were investigated by molecular docking and residue free energy decomposition calculations. Figure 7 illustrates the residues with contribution values more negative than −1 kcal/mol. Structurally, according to the different parent nucleus, these four hit compounds can be classified into two types. One is **Comp2**, belonging to the amide compound; the other is **Comp3**, **Comp4**, and **Comp7**, all belonging to 1,2,4-triazolo-4,3-α-quinazoline compounds.

**Figure 8:**
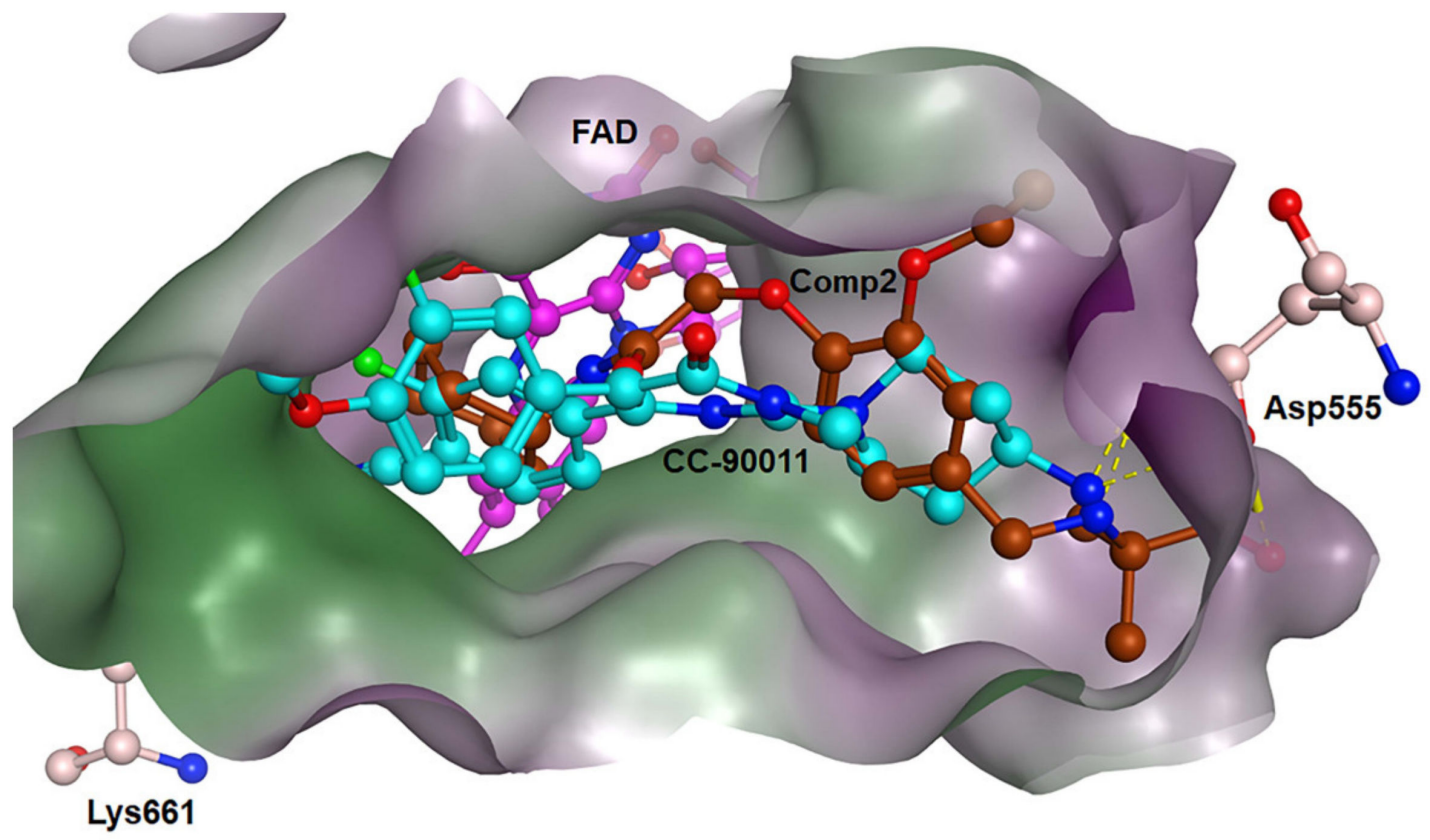
Binding modes of LSD1 with **CC-90011** (blue) and **Comp2** (brown).

For **Comp2**, the electronegative residue Asp555 has an outstanding contribution to the interaction, with a decomposition energy of −3.53 kcal/mol (Table S8). The hydroxyl and nitrogen atoms on the side chain of Asp555 form a relatively strong hydrogen bond with the carboxyl oxygen atom of **Comp2**, which likely facilitates the binding orientation and helps reduce off-target effects (Figure 8). Except this, residue Thr810, Tyr761, and Ala809 are involved in hydrophobic interaction with **Comp2** (Table S9). Additionally, co-factor **FAD** also has a great contribution to the interaction, with a decomposition energy of −3.35 kcal/mol, which is only slightly lower than the largest contributor Asp555 (Table S8). It was found that **FAD** plays an essential role in maintaining the stability of **Comp2** in the targeted substrate pocket through hydrophobic interactions, accounting for 95.76% of the hydrophobic fraction (Table S9).

**Figure 9:**
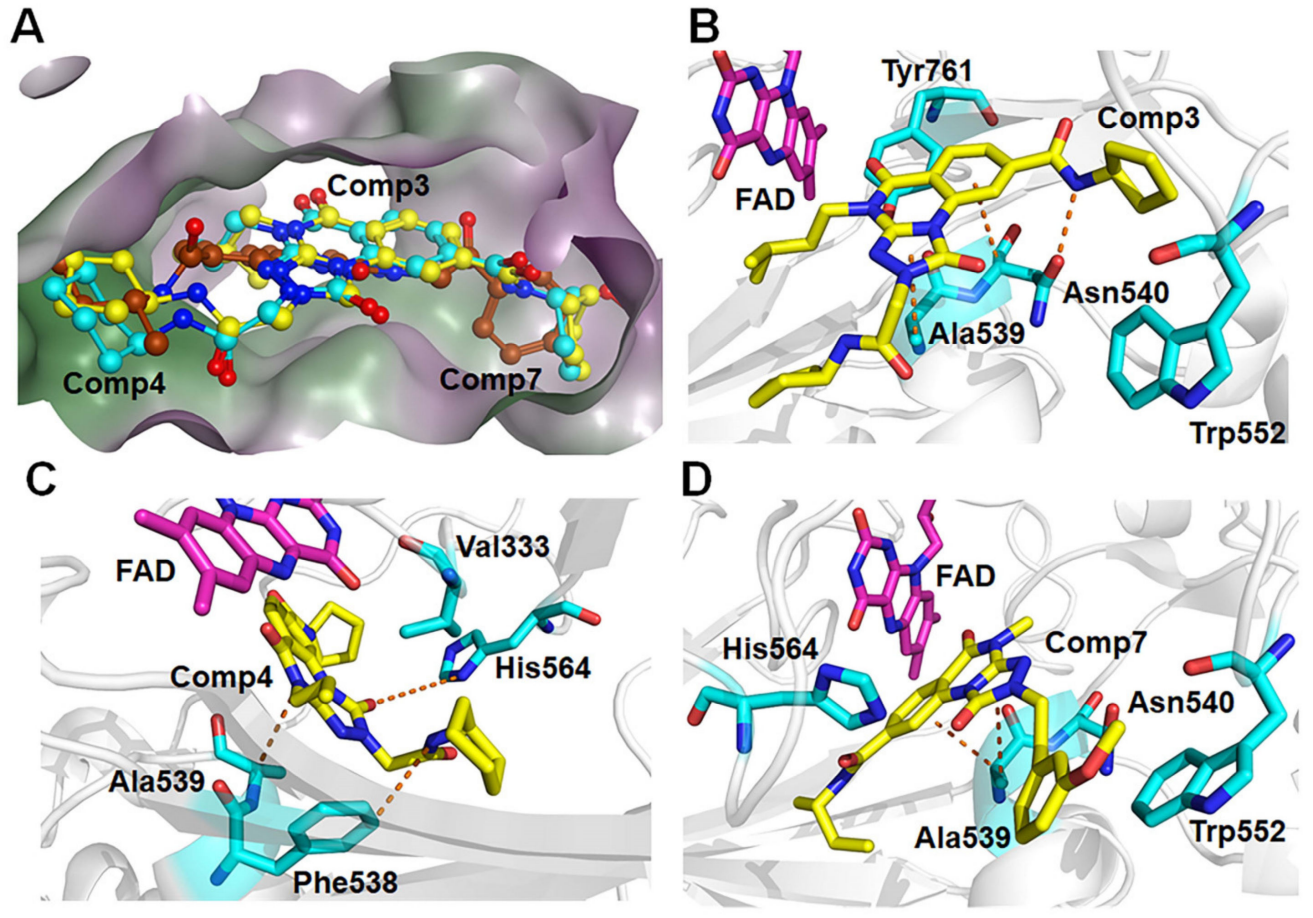
(**A**) The aligned conformations of **Comp3** (yellow), **Comp4** (blue), and **Comp7** (brown). Schematic diagram of modes of LSD1 with (**B**) **Comp3**, (**C**) **Comp4**, and (**D**) **Comp7**.

For the other type, the docking conformations superposition diagram of these three hit compounds in the binding pocket (Figure 9A) reveals that their parent nucleus overlaid well. For **Comp3**, residue Asn540 and Ala539 give the greatest favorable contributions to their binding (−4.46 kcal/mol and −3.00 kcal/mol), which also opportunely form Pi-H interactions with the benzene ring and 1,2,4-triazole ring of parent nucleus, respectively (Figure 9B). Additionally, hydrogen bond analysis shows that the oxygen atom of Asn540 can also form a hydrogen bond with the nitrogen atom on the amide of **Comp3**, indicating this bond made the function to orient and tighten the binding affinity to a large certain (Figure 9B).

**Comp4** exhibits the highest binding affinity with LSD1 among the hit compounds. The contributions of residues are quite average, with no particular residue standing out. There are five residues whose decomposition energies are more negative than −2 kcal/mol, including Ala539, His564, Val333, Phe538, and Lys661. Similar to **Comp3**, the pyrimidine ring of **Comp4** interacts with the main contributor Ala539 through Pi-H interaction, which shows the highest decomposition energy of −2.52 kcal/mol (Figure 9C and Table S8). Furthermore, Phe538, Ala539, and Val333, all of which are hydrophobic residues, contribute to the binding affinity through hydrophobic interactions, with hydrophobic occupancies of 95.62%, 66.41%, and 62.74%, respectively (Table S9). The electropositive residue His564 forms hydrogen bonds with **Comp4** through its nitrogen atoms on the ring, with a decomposition energy of −2.34 kcal/mol (Figure 9C and Table S8). **Comp4** additionally forms hydrophobic interactions with **FAD**, with a decomposition energy of −2.08 kcal/mol, implying the essential role of **FAD** in stabilizing **Comp4** and reducing off-target effects (Table S8).

Regarding **Comp7**, it has the least and relatively weaker residue contributors overall. Among the contributors, Ala539 and Asn540 contribute the most (−3.57 kcal/mol and −2.70 kcal/mol). Specifically, Ala539 forms Pi-H interactions with the benzene ring and 1,2,4-triazole ring of **Comp7**, respectively (Figure 9D). It also has less hydrophobic interaction with **FAD** compared to **CC-90011**. This may explain why **Comp7** has the lowest ∆G_bind_ among the three hit compounds. Therefore, the differences in binding affinity mainly originate from the branched functional groups.

## 3. Materials and Methods

### 3.1. Database Preparation

Commercial databases derived from Taosu biochemical company, including Chemdiv, Maybridge, Lifechemical, Bionet, Specs, and Enamine, were selected for both SBVS and LBVS, with a total of 2,029,554 small molecules. The Wash module of MOE2020 (Chemical Computing Group Inc., Montreal, QC, Canada) software was utilized to optimize all small compounds, including adding hydrogen atoms, 3D structure conversion, and AM1-BCC charge fitting using the Amber10:EHT force field. Lipinski’sand Veber’s rules [32,33] were used to filter out drug-likeness molecules.

### 3.2. Structure-Based Virtual Screening (SBVS)

#### 3.2.1. The Binding Site of LSD1 Receptor

The LSD1 receptor structure was obtained from the co-crystal structure of LSD1 with **CC-90011** (PDB ID: 6W4K) [26] in the RCSB protein data bank (https://www.rcsb.org/ 1 March 2021). The LSD1 protein was pre-processed through residue correction, adding hydrogen at pH 7.0, and charge fitting by both Schrödinger software [34] and AutoDock Tools software [35] (ADT, version 1.5.6). As reported [26], the active substrate-binding pocket of LSD1 is located on the surface of the AOL structure domain, comprising 22 residues, including Gly330, Met332, Val333, Thr335, Ile356, Gln358, Phe538, Ala539, Asn540, Trp552, Asp555, His564, Lys661, Leu659, Leu677, Trp695, Leu706, Tyr761, Ser762, Pro808, Ala809, and Thr810.

#### 3.2.2. Molecular Docking Study in SBVS

A multi-stage and progressive approach was adopted for the SBVS of LSD1 inhibitors, which consists of three screening rounds completed jointly by Schrödinger and AutoDock Vina. The first two rounds employ high-throughput virtual screening (HTVS) and standard precision (SP) modules in Schrödinger software. In the first screening round, known for its relatively low discrimination and time-saving character, the HTVS method can quickly confirm whether small molecules can enter the active site of protein or not, filtering out compounds with poor matching degree and low affinity. Only the top-scoring compounds (top 50%) were retained. In the second screening round, the SP method (GlideScore function) was applied to re-dock these retained compounds to the docking site, and the top-scoring compounds (top 30%) were subsequently selected. The LigPrep module was used in both screening rounds mainly to generate all tautomers of small molecules, and the Receptor grid generation module was used to generate grid points. A docking box was then generated at the primordial ligand site of the protein, and molecular docking was performed using the Glide docking module under default parameters. In the third screening round, the extra precision (XP) docking module of Schrödinger with EmodelSore function and the semi-flexible docking method in Autodock Vina with Vina scoring function were adopted, respectively. In Autodock Vina, the docking box size was set as 15 Å × 15 Å × 15 Å, and the exhaustiveness was set to 24. Finally, the top-scoring compounds (top 10%) were selected, and their intersection was taken, considering the scoring function and matching degree with the active pocket.

### 3.3. Ligand-Based Virtual Screening

#### 3.3.1. Training Set and Test Set Preparation

In order to establish a reliable ligand-based pharmacophore model, 45 reported LSD1 inhibitors [19,21,22,23,24,26,36,37,38,39,40,41] targeting the substrate-binding pocket were selected based on the following protocol. These selected inhibitors with similar activity values were required to maintain structural diversity, and their activities should span at least four orders of magnitude. Among them, 21 diverse compounds (Table S1) were selected as the training set, and the remaining 24 compounds (Table S2) were classified into the test set for validating the pharmacophore model. The IC_50_ values of the compound in the training and the test set ranged from 0.25 nM to 16,000 nM and from 0.70 nM to 33,900 nM, respectively. Structural energy minimization was performed using the CHARMM [42] force field to obtain the dominant conformations. These prepared compounds were used for pharmacophore generation and validation by DS2017R2 software (Accelrys Software Inc., San Diego, CA, USA).

#### 3.3.2. Pharmacophore Model Generation

Utilizing the Catalyst HypoGen algorithm, a set of ligands with known activity values was used to derive corresponding hypothesis models [43]. This algorithm is devoted to finding common hypotheses among the active compounds in the training set, constructing a pharmacophore model that correlates best with the measured activities while using as few features as possible [44]. In order to effectively correlate the training set with their activities, the uncertain value was set to 1.5. The conformation generation method was set to Fast with an energy threshold of 10 kcal/mol, and each molecule generated a maximum of 255 diverse conformations. In this algorithm, all conformational models were used for each compound in the training set, not just the lowest energy conformation. Then, five features, namely hydrophobic (HY), ring aromatic (RA), positive ionizable center (PI), hydrogen bond acceptor (HBA), and hydrogen bond donor (HBD), were selected to generate feature mappings. Considering the spatial effects of compounds, the maximum excluded volume was set, and the minimum interfeature distance was set to 1.5. Finally, 10 pharmacophore models with significant statistical parameters were generated.

#### 3.3.3. Pharmacophore Model Validation

After generating the pharmacophore from the training set, three methods were adopted to validate its accuracy, including cost analysis, test set validation, and Fischer’s randomization test.

##### Cost Analysis

A cost analysis was performed to evaluate the complexity, chemical characteristics, and errors of the generated pharmacophore model compared to the experimental activity. In the standard HypoGen model, the configuration should not exceed 17. The total cost, fixed cost, and null cost represent the actual cost of the pharmacophore, the cost of a perfect pharmacophore, and the cost of the worst possible pharmacophore, respectively. The difference between total cost and null cost should be maximized. A difference greater than 60 bits suggests a true correlation in the used data set exceeding 90% [31]. The total cost of any hypothesis should be close to the fixed cost and away from the null cost. Meanwhile, the root mean squared (RMS) represents the correlation (Correl) quality between the estimated and actual activity. So, high-quality models should have the highest activity value correlation coefficient and the lowest RMS value [45].

##### Test Set Validation

This hypothesis aimed to identify the active structures and predict the activities of molecules. To validate the capability of the pharmacophore hypothesis, 24 compounds with different structures in the test set were applied, and their activities were predicted. The model was evaluated according to the correlation coefficient q^2^ of the linear regression used to calculate log (activities) values. All queries were performed using the Ligand Pharmacophore Profiler protocol in DS2017R2 as well.

##### Fischer’s Randomization Test

Fischer’s randomization test method was applied to assess the statistical significance of the best hypotheses by confirming the strong correlation between chemical structures and biological activities. In this technique, the activity value was randomly assigned to the compounds in the training set on the premise of a confidence level set at 95%. Then, 19 randomized training sets were generated and utilized to create 19 random pharmacophores by HypoGen algorithm using identical features and parameters. If these pharmacophores showed similar or superior statistics, the original hypothesis was deemed not to be generated by chance [46,47].

### 3.4. Molecular Docking Study in LBVS

Molecular dockings between LSD1 and the compounds that passed pharmacophore matching and satisfied Lipinski’s and Veber’s rules were performed using AutoDock Vina program. The main parameters used here were identical to those used in the SBVS module.

### 3.5. ADMET Screening

ADMET screening was assessed using DS2017R2 software and ADMETlab2.0 server [48] for SBVS and LBVS, respectively, to reduce drug development costs. The primary descriptors for ADMET prediction in SBVS included aqueous solubility, blood–brain barrier penetration (BBB), human intestinal absorption (HIA), ames mutagenicity (AMES), weight-of-evidence rodent, and carcinogenicity (WOE). In LBVS, the chosen parameters included BBB, HIA, Caco-2 permeability, madin−darby canine kidney (MDCK) permeability, plasma protein binding (PPB), hERG, AMES, human hepatotoxicity (H-HT), and rat oral acute toxicity (ROA). Finally, the Pan Assay Interference Compounds (PAINS) [49] filter was applied in ADMETlab2.0 to eliminate false-positive molecules.

### 3.6. Molecular Dynamics Simulations

Taking the best-docked receptor-ligand complexes as the initial structure models, all MD simulations were performed using the GPU-accelerated version of the PMEMD [50,51] module in Amber18 packages [52]. All hit compounds were firstly optimized at B3LYP/6-31G (d, p) level using Gaussian16 [53]. The general AMBER force field (GAFF2) [54] and the AMBER ff14SB [55] force field were applied to the hit compounds and protein, respectively. Four chloride counterions were added to each system to maintain the electro-neutrality. All studied systems were immersed in a TIP3P [56] water box, with a minimum distance of 12 Å between the box edges and any protein/substrate atom.

The initial models were minimized using a combination of 80,000 steps of steepest descent and 20,000 steps of conjugate gradients methods. Subsequently, the system was gradually heated from 10 K to 300 K using Langevin dynamics [57,58] with a collision frequency of 5.0 ps^−1^. Then, the system was equilibrated for 10 ns in the NPT ensemble at 300 K and 1 bar. Anisotropic pressure regulation was performed using the Berendsen barostat with a pressure relaxation time of 2.0 ps. Finally, 175 ns and 200 ns production MD simulations were performed for SBVS and LBVS, respectively. Periodic boundary conditions were employed, and the long-range electrostatic interactions were described using the particle mesh Ewald method (PME) [59]. A cut-off radius of 8 Å was used for evaluating short-range interactions. The SHAKE algorithm [60] was used to constrain chemical bonds containing H atoms, with a constraint constant of 10^−6^ and a time step of 2 fs. The equilibrated portion of the trajectories was analyzed using AmberTools CPPTRAJ program [61].

### 3.7. Binding Free Energy Calculations

Considering a good balance between computational efficiency and accuracy, the MM/PBSA method was finally chosen to calculate protein–ligand binding free energy through the MMPBSA.py [62] script of Amber18 package. First, 5000 snapshots extracted from the last 50 ns trajectories at a 20 ps interval were used to calculate the binding free energy. The ionic strength was set to 0.15 M, and the external and internal dielectric constants were set to 80 and 4, respectively.

The binding free energy (∆G_bind_) was computed by the following equation, where ∆G_complex_, ∆G_receptor_, and ∆G_ligand_ were the free energies of the complex, receptor, and ligand, respectively.

ΔG_bind_ = ΔH − TΔS = ΔE_MM_ + ΔG_solv_ − TΔS (1)

ΔE_MM_ = ΔE_ele_ + ΔE_vdw_ + ΔE_int_ (2)

ΔG_solv_ = ΔG_PB_ + ΔG_nonp_ (3)

Here, −T∆S represented entropy contribution. Since entropic calculation is time-consuming, and its value will fluctuate if a small number of snapshots are adopted, it was omitted in this study [63]. ∆E_gas_ is gas phase molecular mechanical energy, and ∆G_solv_ is solvation free energy. ∆E_int_, ∆E_vdw_, and ∆E_ele_ are the internal energy, van der Waals interaction energy, and electrostatic energy, respectively. The total value of ∆E_int_ accounting for bond, angle, and dihedral energies was set to zero. The ∆G_solv_ was calculated as the sum of the polar contribution (∆G_PB_) to the solvation free energy calculated by the Poisson–Boltzmann (PB) model and non-polar contribution (∆G_nonp_) derived from a linear relation to the solvent-accessible surface area (SASA).

ΔG_nonp_ = γ SASA + β (4)

The empirical constants γ and β were set as 0.00542 kcal/mol/Å^2^ and 0.92 kcal/mol, respectively. The SASA was determined by a probe radius of 1.4 Å. To further explore the detailed interaction information of complex, residue free energy decomposition was performed by the MM/PBSA method to identify the key residues responsible for binding free energy. The contribution of each residue was calculated without considering the contribution of entropies. The contribution was defined as the sum of ∆E_vdw_, ∆E_ele_, ∆G_PB_, and ∆G_nonp_:

∆G_residue_ = ∆E_vdw_ + ∆E_ele_ + ∆G_PB_ + ∆G_nonp_ (5)

## 4. Conclusions

LSD1 plays an important role in the occurrence and development of various tumors and has recently become a new molecular target for tumor prevention and treatment. Thus, designing high-efficiency and low-toxicity LSD1 inhibitors with novel structures started to cause more and more interest of pharmaceutical chemists. In this work, we systematically searched for reversible LSD1 inhibitors that targeted the substrate-binding pocket using computer-aided drug design technology. Mainly, structure-based and ligand-based virtual screenings were employed to further identify potential novel structures using molecular docking and pharmacophore model strategies, respectively. Through drug-likeness evaluation, ADMET screening, MD simulations, and binding free energy-based screening, one and four hit compounds were finally screened out from the databases (2,029,554 compounds). The hit compound **Lig2** obtained through SBVS belongs to amide, while the four compounds obtained through LBVS can be divided into two groups according to the parent nucleus. One belongs to the amide compound (**Comp2**), and the other is 1,2,4-triazolo-4,3-α-quinazoline (**Comp3**, **Comp4**, **Comp7**). Among them, **Comp4** exhibits the strongest binding affinity. Finally, the binding mechanisms of these hit compounds were further calculated in detail by residue free energy decomposition. It is indicated that van der Waals force is the largest contributor to the binding affinity contribution of all hit compounds to LSD1, and **FAD** is also helpful in stabilizing the binding and avoiding off-target effects. In future, it would be feasible to perform further structure optimizations based on these novel hit compounds to improve the bioactivity continuously. Our work expands the diversity of parent nucleus of LSD1 substrate reversible inhibitors and provides a solid theoretical foundation for the design and further optimization of synthetic chemists.

## Figures and Tables

**Table 1:** Compounds obtained after visualized analysis through SBVS.

Ligand	Structure	Ligand	Structure
**1**	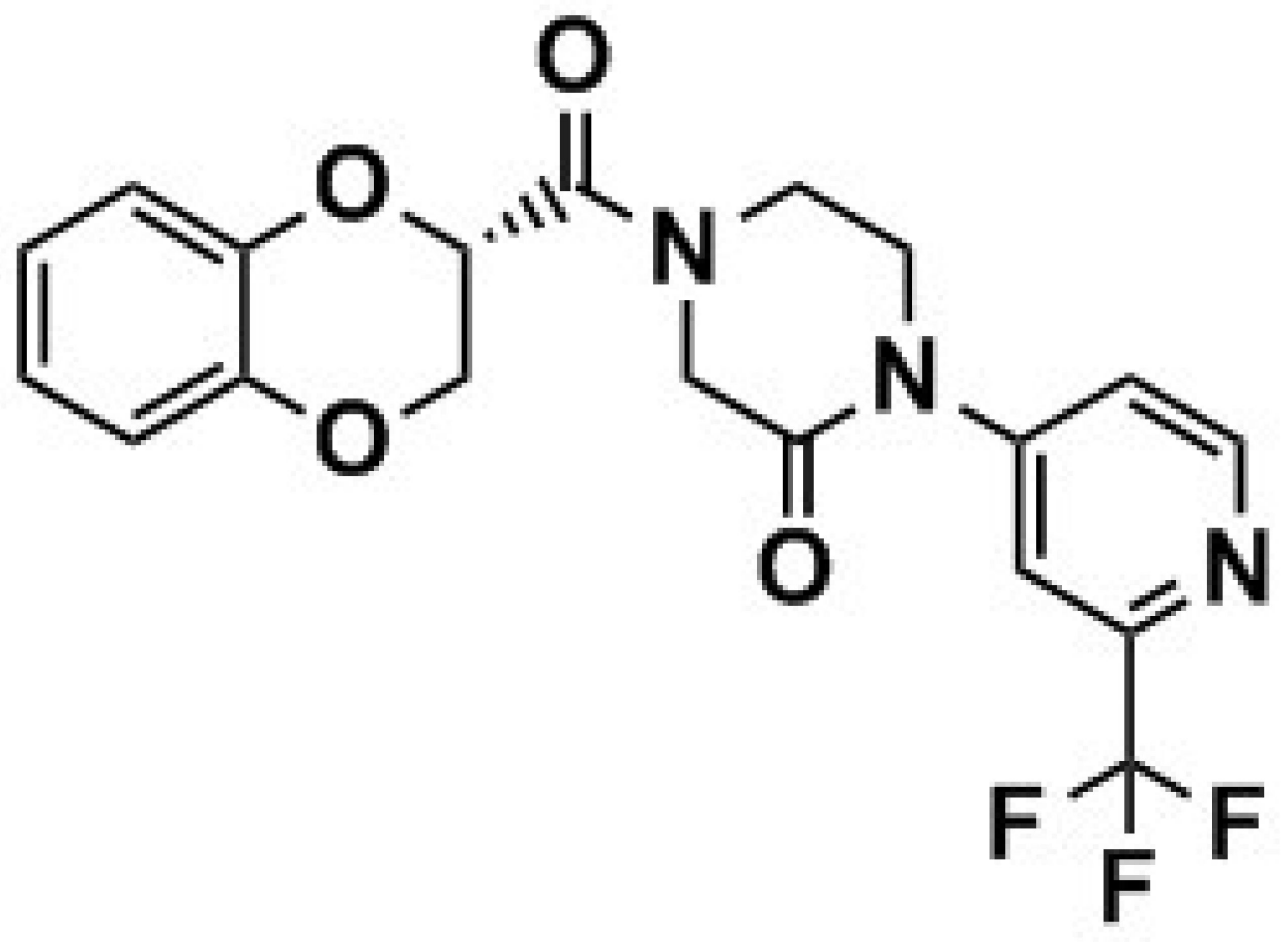	**4**	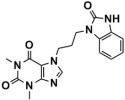
**2**	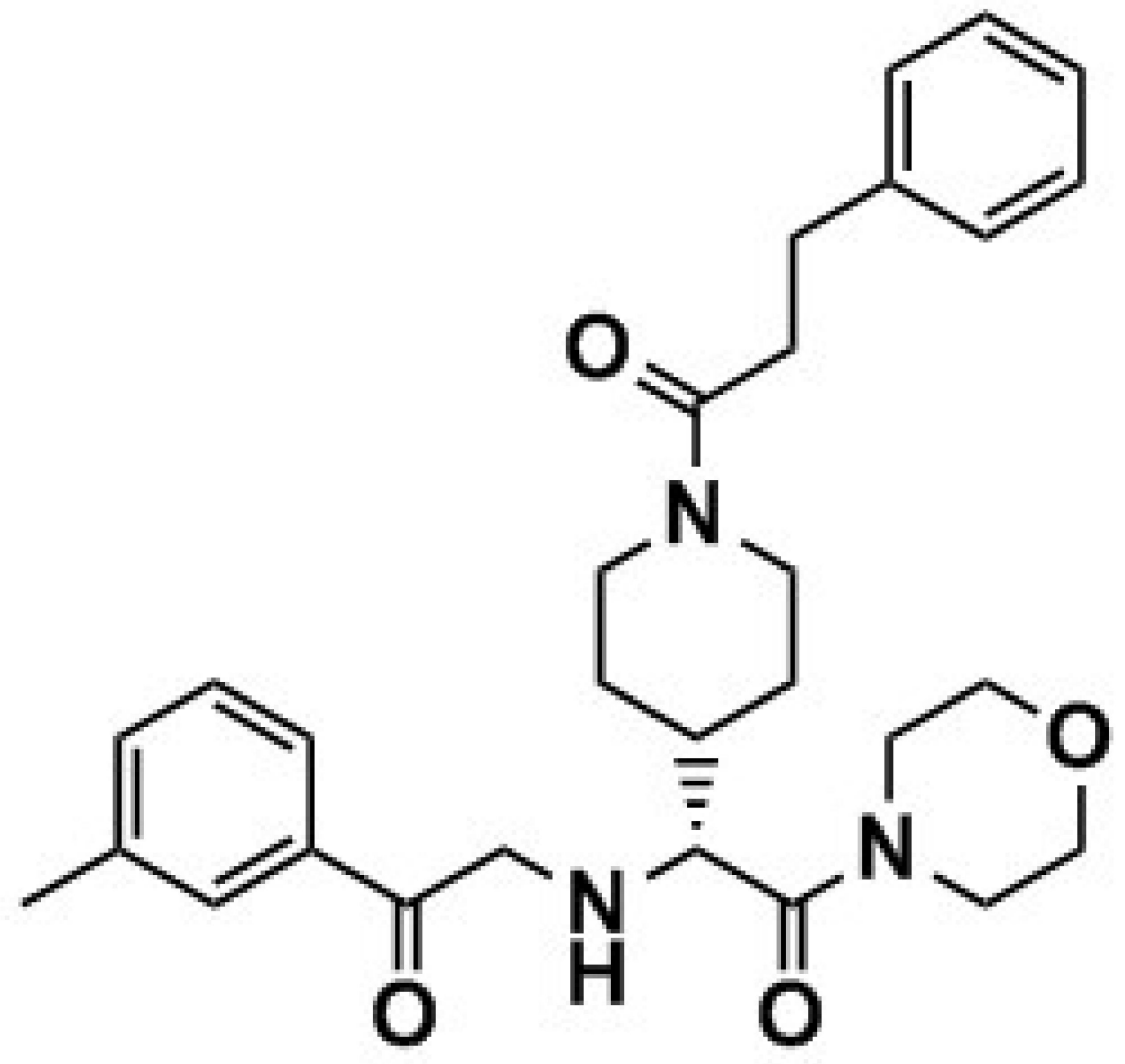	**5**	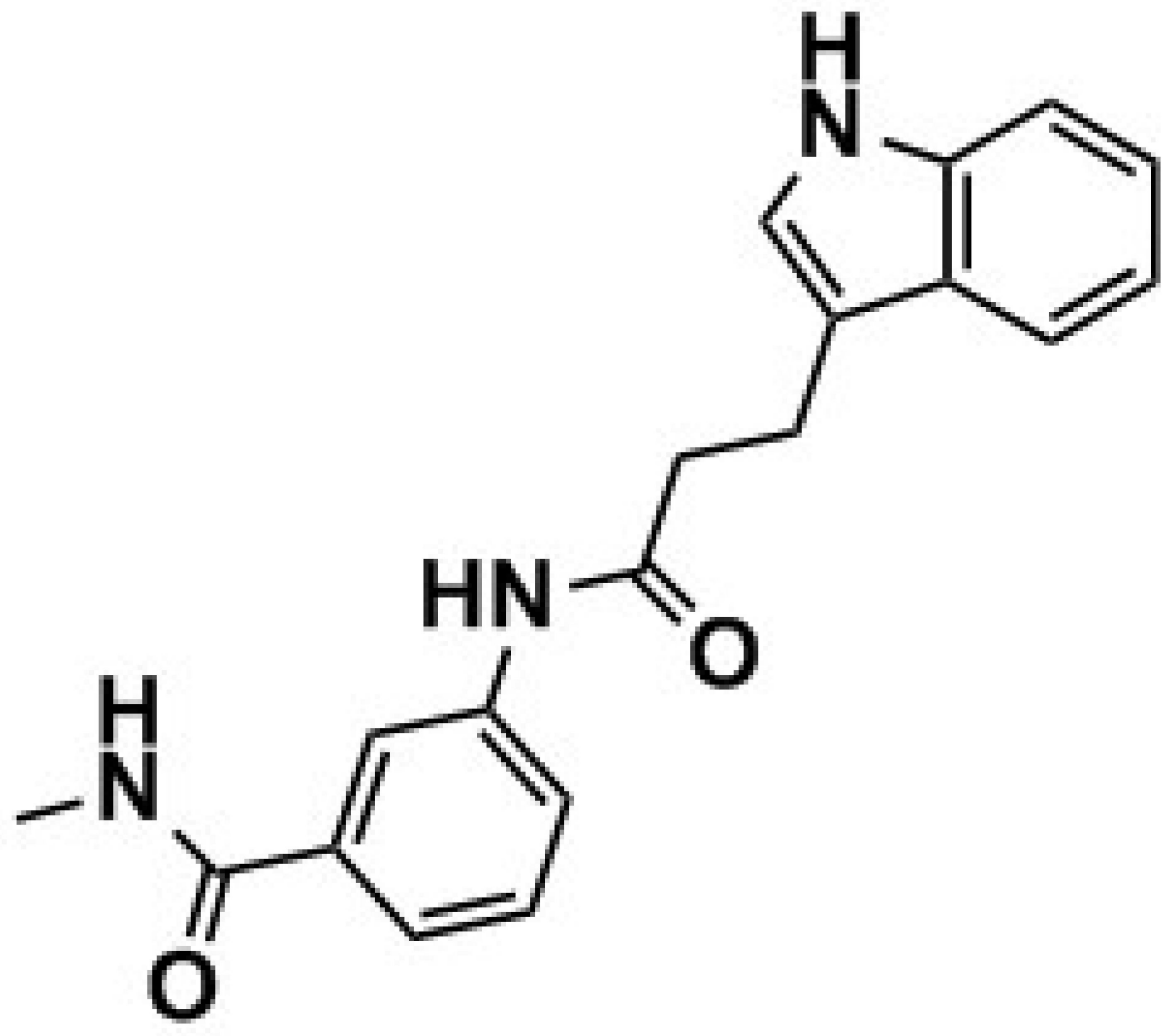
**3**	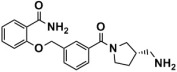	**6**	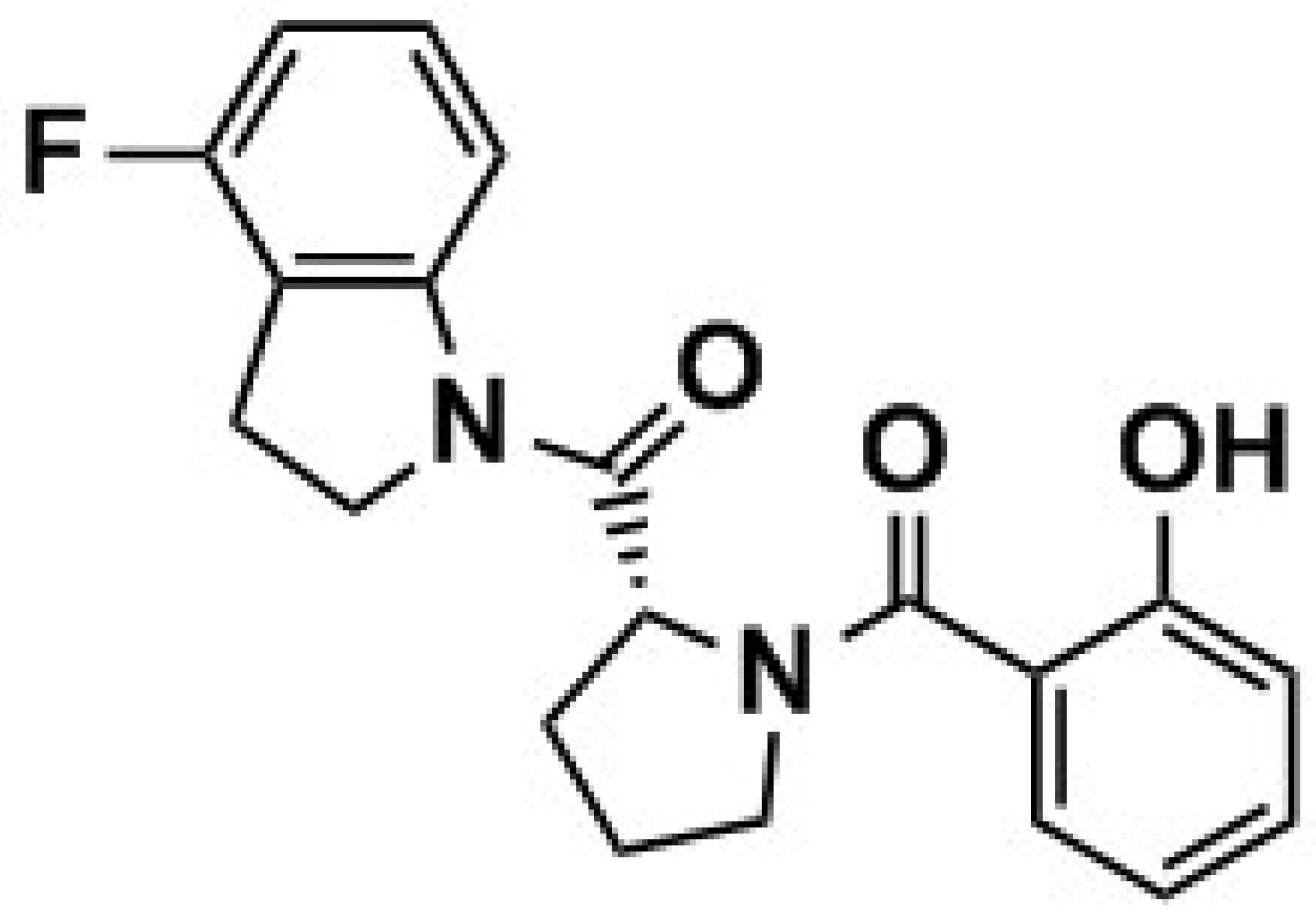

**Table 2:** Calculated MM/PBSA binding free energy of the MD simulations through SBVS.

Ligand	ΔE_vdw_	ΔE_ele_	ΔG_PB_	ΔG_nonp_	ΔG_bind_
**CC-90011**	−44.82	−7.38	12.30	−4.53	−44.42
	(±0.03)	(±0.03)	(±0.04)		(±0.03)
**1**	−40.57	−6.67	13.57	−4.41	−38.09
	(±0.05)	(±0.02)	(±0.02)		(±0.04)
**2**	−65.35	−5.97	13.92	−5.73	−63.13
	(±0.04)	(±0.01)	(±0.01)		(±0.04)
**3**	−35.60	−3.48	9.43	−3.50	−33.15
	(±0.04)	(±0.03)	(±0.03)		(±0.04)
**4**	−36.74	−8.54	13.26	−3.90	−35.92
	(±0.05)	(±0.02)	(±0.02)		(±0.05)
**5**	−34.05	−7.71	12.54	−3.80	−33.03
	(±0.05)	(±0.03)	(±0.02)		(±0.04)
**6**	−25.96	−6.21	8.35	−3.11	−26.95
	(±0.07)	(±0.05)	(±0.04)		(±0.06)

Unit: kcal/mol; ΔE_ele_, ΔE_vdw_, ΔG_PB_, and ΔG_nonp_ represent the electrostatic energy, van der Waals energy, polar solvation energy and non-polar solvation energy, respectively.

**Table 3:** Characteristics of ten hypotheses for the training set generated by HypoGen algorithm.

Hypothesis	Total Cost	Cost Difference ^1^	RMS	Correlation	Maximum Fit	Features ^2^
**1**	97.64	588.12	1.68	0.98	10.72	HBD, RA, HY, PI
**2**	103.83	581.93	1.86	0.97	10.36	HBD, RA, HY, PI
**3**	106.03	579.73	1.92	0.97	10.28	HBD, RA, HY, PI
**4**	108.51	577.25	2.00	0.97	9.69	HBD, RA, HY, PI
**5**	223.17	462.59	3.87	0.87	9.06	RA, HY 2PI
**6**	227.56	458.20	3.93	0.86	8.80	HBD, RA, HY, PI
**7**	236.46	449.30	4.03	0.86	9.26	HBD, 2HY, PI
**8**	237.41	448.35	4.05	0.85	8.40	RA, HY, 2PI
**9**	238.54	447.22	4.05	0.85	9.20	HBD, 2HY, PI
**10**	242.37	443.39	4.11	0.85	7.90	2HY, 2PI,

^1^ Cost Difference = (null cost − total cost). The null cost, fixed cost, and configuration cost values were 685.76, 65.47, and 14.64, respectively. All costs were in bit units. ^2^ HBD: hydrogen bond donor, HY: hydrophobic, PI: positive ionizable, RA: ring aromatic.

**Table 4:** Experimental and estimated activities of 21 compounds based on HypoGen1 in the training set.

Compound	Experimental IC_50_ (nM)	Estimated IC_50_ (nM)	Error ^1^	Experimental Scale ^2^	Estimated Scale ^2^	Fit Value ^3^
**1**	0.25	0.26	1.03	+++	+++	9.71
**2**	0.50	0.27	−1.82	+++	+++	9.68
**3**	7.80	27.06	3.47	+++	+++	7.69
**4**	13.00	26.91	2.10	+++	+++	7.69
**5**	57.00	53.42	−1.10	+++	+++	7.39
**6**	79.00	109.01	1.38	+++	+++	7.09
**7**	123.00	149.26	1.21	+++	+++	6.95
**8**	210.00	74.12	−2.83	++	+++	7.25
**9**	230.00	169.50	−1.36	++	+++	6.89
**10**	540.00	745.47	1.38	++	++	6.25
**11**	804.00	466.07	−1.73	++	++	6.45
**12**	1850.00	5810.45	3.14	++	+	5.36
**13**	2200.00	5813.18	2.64	+	+	5.36
**14**	2800.00	5201.87	1.86	+	+	5.41
**15**	2900.00	5814.20	2.00	+	+	5.36
**16**	4000.00	1797.15	−2.23	+	++	5.87
**17**	8130.00	5840.49	−1.40	+	+	5.36
**18**	9500.00	5811.38	−1.63	+	+	5.36
**19**	9600.00	5853.05	−1.64	+	+	5.36
**20**	13,900.00	5811.33	−2.40	+	+	5.36
**21**	16,000.00	5810.56	−2.75	+	+	5.36

^1^ Difference between the predicted and experimental activity values. ^2^ Most active (<200 nM, +++), moderately active (200–2000 nM, ++), and inactive (>2000 nM, +). ^3^ Fit value indicates how well the features in the pharmacophore overlap the chemical features in the molecule. Fit = weight × [max (0.1 − SSE)] where SSE = (D/T)2, D = displacement of the feature from the center of the location. Constraints and T = the radius of the location constraint sphere for the feature (tolerance).

**Table 5:** Compounds obtained after ADMET analysis through LBVS.

Compound	Structure	Compound	Structure
**1**	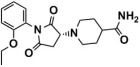	**5**	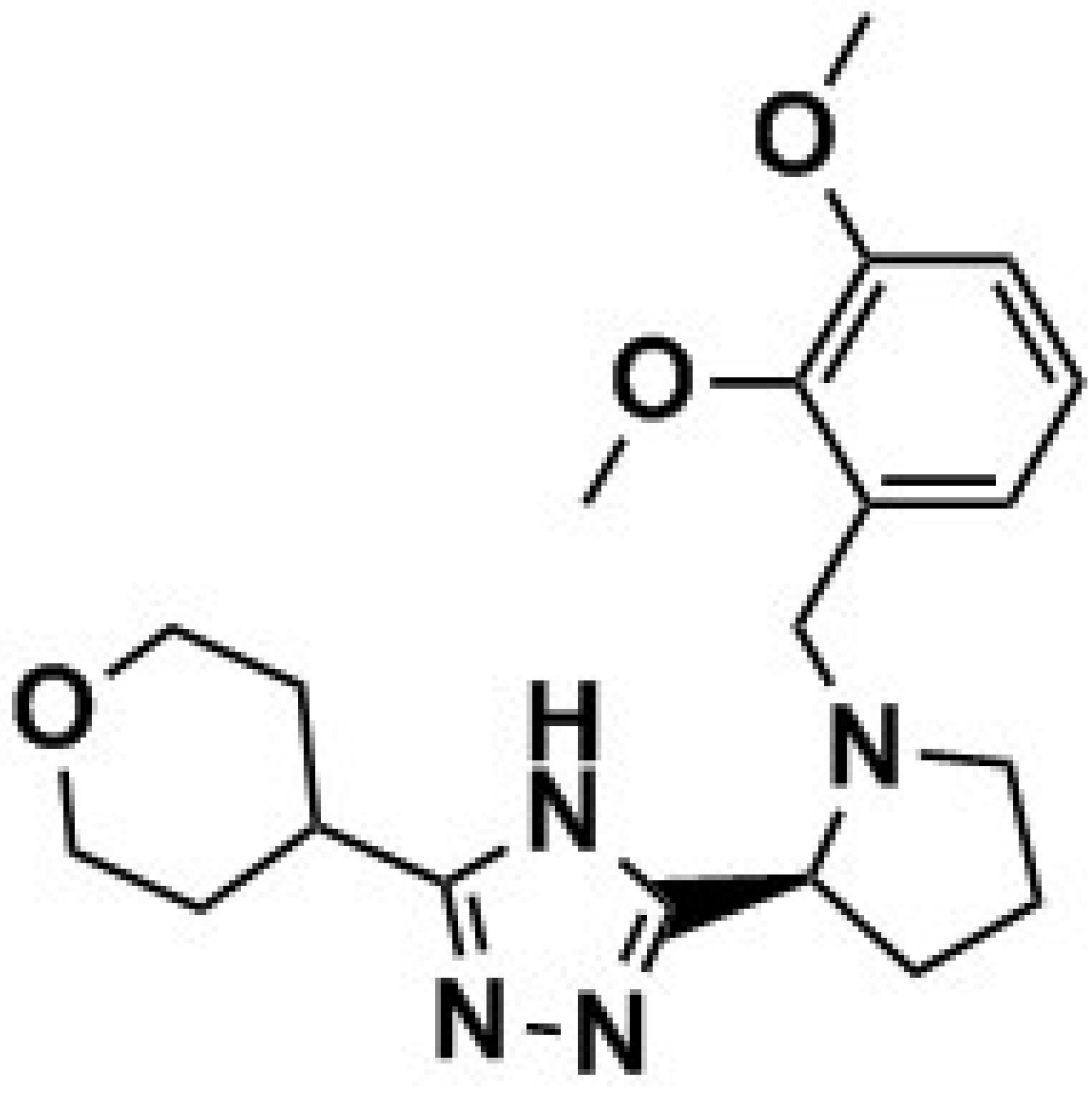
**2**	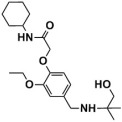	**6**	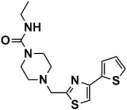
**3**	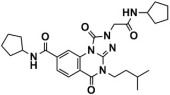	**7**	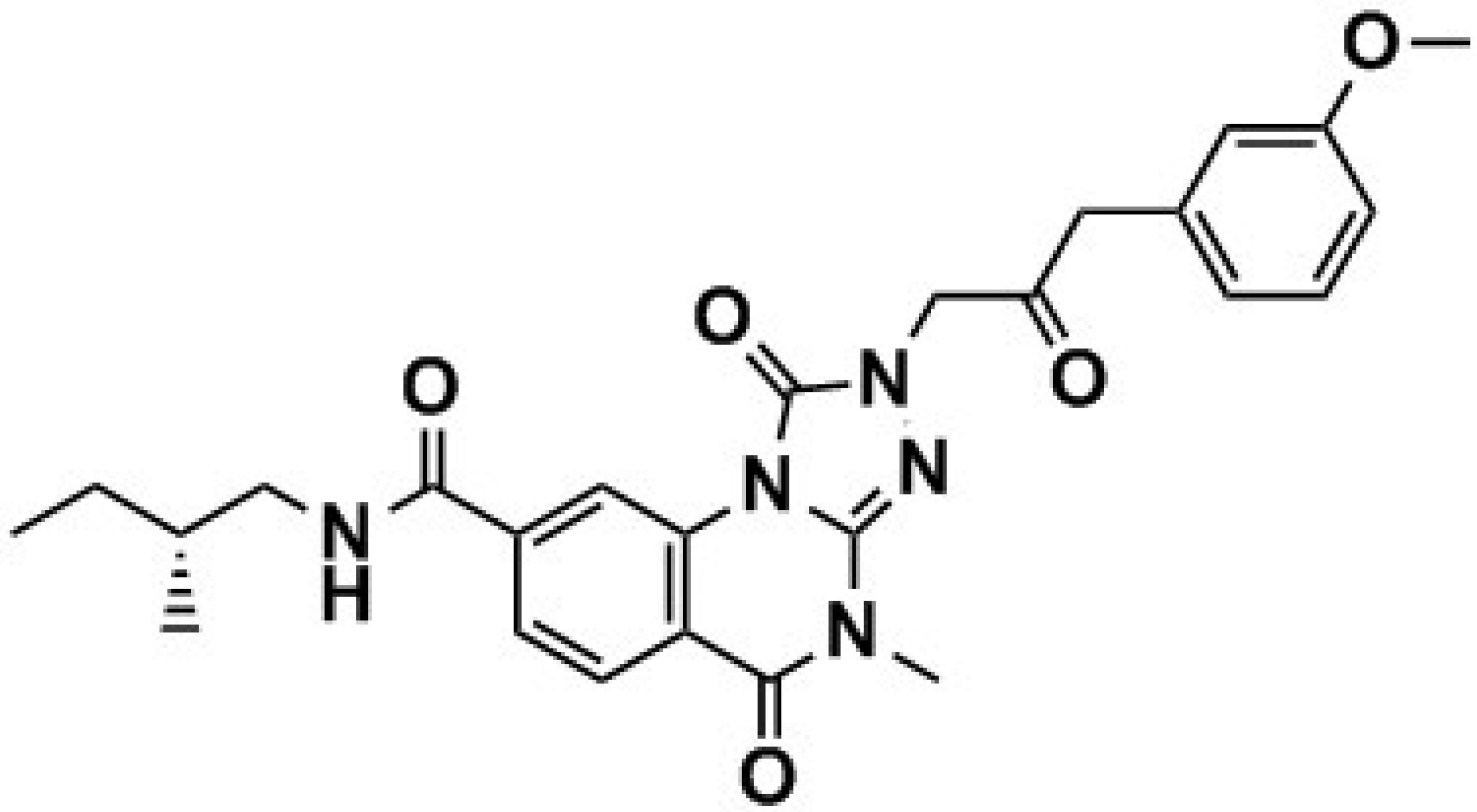
**4**	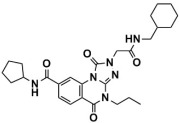		

Unit: kcal/mol; ΔE_ele_, ΔE_vdw_, ΔG_PB,_ and ΔG_nonp_ represented the electrostatic energy, van der Waals energy, polar solvation energy and non-polar solvation energy, respectively.

**Table 6:** Calculated MM/PBSA binding free energy of the MD simulations through LBVS.

Compound	ΔE_vdw_	ΔE_ele_	ΔG_PB_	ΔG_nonp_	ΔG_bind_
**CC-90011**	−44.82	−7.38	12.30	−4.53	−44.42
	(±0.03)	(±0.03)	(±0.04)		(±0.03)
**1**	−41.84	−6.12	11.09	−4.24	−41.10
	(±0.04)	(±0.03)	(±0.02)		(±0.04)
**2**	−50.32	−6.67	13.33	−4.85	−48.51
	(±0.05)	(±0.03)	(±0.03)		(±0.05)
**3**	−51.50	−5.40	11.17	−5.32	−51.06
	(±0.05)	(±0.04)	(±0.04)		(±0.05)
**4**	−57.03	−1.51	9.90	−5.62	−54.26
	(±0.05)	(±0.03)	(±0.03)		(±0.05)
**5**	−35.49	−6.53	10.41	−4.02	−35.63
	(±0.05)	(±0.02)	(±0.02)		(±0.05)
**6**	−37.26	−2.97	8.17	−4.14	−36.21
	(±0.04)	(±0.02)	(±0.02)		(±0.04)
**7**	−47.85	−5.90	12.53	−5.24	−46.45
	(±0.05)	(±0.02)	(±0.02)		(±0.05)

## Data Availability

Suggested Data Availability Statements are available in section “MDPI Research Data Policies” at https://github.com/bigsmartguy/LSD1-Yang (accessed on 3 April 2023).

## Supplementary Materials

The following supporting information can be downloaded at: https://www.mdpi.com/article/10.3390/molecules28145315/s1, Figure S1: (A) RMSD and (B) RMSF results during the molecular dynamics simulations of sole-LSD1, LSD1_CC-90011, LSD1_Lig1, LSD1_Lig2, LSD1_Lig3, LSD1_Lig4, LSD1_Lig5 and LSD1_Lig6 systems through SBVS; Figure S2: (A) RMSD and (B) RMSF results during the molecular dynamics simulations of sole-LSD1, LSD1_CC-90011, LSD1_Comp1, LSD1_Comp2, LSD1_Comp3, LSD1_Comp4, LSD1_Comp5, LSD1_Comp6 and LSD1_Comp7 systems through LBVS; Table S1: The smile formats and experimental IC50 values of 21 compounds in the training set; Table S2: The smile formats and experimental IC50 values of 24 compounds in the test set. Table S3: The drug-likeness and ADMET properties of compounds screened after visualized analysis through SBVS; Table S4: Calculated RMSD values of complexes in the MD simulations through SBVS and LBVS; Table S5: Free energy decomposition calculations for the binding pocket-composed residues in LSD1_CC-90011 and LSD1_Lig2 complexes through SBVS; Table S6: Experimental and estimated activities of 24 compounds based on HypoGen1 in the test set; Table S7: The drug-likeness and ADMET properties of compounds screened after ADMET analysis through LBVS; Table S8: Free energy decomposition calculations for the binding pocket-composed residues in LSD1_Comp2, LSD1_Comp3, LSD1_Comp4 and LSD1_Comp7 complexes through LBVS; Table S9: Hydrophobic interaction analysis in **LSD1_Comp2** and **LSD1_Comp4** complexes through LBVS.

## Sample Availability

Samples of the compounds are not available from the authors.
