# Supplementary material for: LSD1-Based Reversible Inhibitors Virtual Screening and Binding Mechanism Computational Study
Source: Molecules. 2023 Jul 10;28(14):5315. doi: 10.3390/molecules28145315 (PMC10383809; doi:10.3390/molecules28145315)

Table S1. The smile formats and experimental IC_{50} values of 21 compounds in the training set.

Compound	Smile formats	IC_{50} (nM)
**1**	COc1ccc(-c2c(-c3ccc(C#N)c(F)c3)nc(N3CCC(N)CC3)n(C)c2=O)cc1F	**0.25**
**2**	Cc1ccc(-c2c(-c3ccc(C#N)c(F)c3)nc(N3CCC(N)CC3)c(=O)n2C)cc1	**0.5**
**3**	C/C(=N\NC(=O)c1cccc(S(=O)(=O)N2CCN(C)CC2)c1)c1cc(Cl)ccc1O	**7.8**
**4**	CCOCc1cccc(NC(=O)c2cc3sccc3n2C)c1COc1ccc(OC[C@H]2CCNC2)cc1	**13**
**5**	Cc1ccc([C@@H]2CN(C(=O)[C@H]3CC[C@H](CN)CC3)C[C@H]2c2ccc(C#N)cc2)cc1	**57**
**6**	COc1ccc(CN(CC(=O)N2CCC(CN)CC2)c2ccc(C#N)cc2)cc1F	**79**
**7**	N#Cc1ccc(-n2nc(C(=O)N3CCC[C@@H](N)C3)cc2OCc2ccccn2)cc1	**123**
**8**	CCc1cc(COc2cc(C(=O)N3CCC[C@@H](N)C3)nn2-c2ccc(C#N)cc2)on1	**210**
**9**	N/C(=N\O)c1ccc(/C=C/c2cc(O)c(O)cc2Br)cc1	**230**
**10**	Cn1c(C(=O)Nc2cccc3c2N(Cc2ccc(OC4CCNCC4)cc2)CCC3)cc2cc(F)ccc21	**540**
**11**	COCc1cccc(NC(=O)c2cc3ccccc3n2C)c1COc1ccc(OC2CCNCC2)cc1	**804**
**12**	Cn1c(C(=O)Nc2ccccc2COc2ccc(O[C@H]3CCNC3)cc2)cc2sccc21	**1850**
**13**	CCN1CCN(C(=S)SCC(=O)Nc2ccc(C(=O)/C=C/c3ccccc3)cc2)CC1	**2220**
**14**	C[C@@H](Oc1cc(C(=O)N2CCC[C@@H](N)C2)nn1-c1ccc(C#N)cc1)C(N)=O	**2800**
**15**	CN(CNC(=O)c1cccc(S(=O)(=O)N2CCOCC2)c1)c1cc(Cl)ccc1O	**2900**
**16**	COc1cc(/C=C/C(=O)CC(=O)/C=C/c2ccc(O)c(OC)c2)ccc1O	**4000**

**17**	COc1ccccc1CNc1n[nH]c(NCc2ccccc2OC)n1	**8130**
**18**	COCc1cccc(NC(=O)c2cc3sccc3n2C)c1	**9500**
**19**	CCc1cn(CCOc2ccccc2)c(-c2cc3sccc3n2C)n1	**9600**
**20**	COc1cc(/C=C/C(=O)Nc2cccc(COc3ccc(OC4CCNCC4)cc3)c2)ccc1O	**13900**
**21**	Cc1cc(NN)n2nc(SCc3ccc(F)cc3)nc2n1	**16000**

Table S2. The smile formats and experimental IC_{50} values of 24 compounds in the test set.

Compound	Smile formats	IC_{50} (nM)
**1**	Cc1ccc(-c2c(-c3ccc(C#N)cc3)nc(N3CCC(N)CC3)c(=O)n2C)cc1	**0.7**
**2**	COc1ccc(-c2c(-c3ccc(C#N)cc3)nc(N3CCC(N)CC3)n(C)c2=O)cc1F	**0.8**
**3**	COc1ccc(-c2c(-c3ccc(C#N)cc3)nc(N3CCC(N)CC3)n(C)c2=O)cc1	**1.4**
**4**	CN1CCN(S(=O)(=O)c2cccc(C(=O)N/N=C3\CCc4c(Cl)ccc(O)c43)c2)CC1	**4.9**
**5**	CN1CCN(S(=O)(=O)c2cccc(C(=O)N/N=C3\CCc4cccc(O)c43)c2)CC1	**9.2**
**6**	N/C(=N\O)c1cccc(/C=C/c2cc(O)c(O)cc2Br)c1	**18**
**7**	Cc1ccc([C@@H]2CN(C(=O)[C@H]3CC[C@@H](CN)CC3)C[C@@H]2c2ccc(C#N)cc2)cc1	**57**
**8**	COCc1cccc(NC(=O)c2cc3sccc3n2C)c1COc1ccc(OC2CCNCC2)cc1	**74.6**
**9**	CCc1cc(COc2cc(C(=O)N3CCC[C@@H](N)C3)nn2-c2ccc(C#N)cc2)on1	**79**
**10**	Cn1c(C(=O)Nc2ccccc2COc2ccc(OC[C@@H]3CCNC3)cc2)cc2sccc21	**85.7**
**11**	Cc1cc(COc2cc(C(=O)N3CCC[C@@H](N)C3)nn2-c2ccc(C#N)cc2)on1	**121**
**12**	COCc1cccc(NC(=O)c2cc3sccc3n2C)c1COc1ccc(OC[C@@H]2CCNC2)cc1	**200**
**13**	Cc1cc(COc2cc(C(=O)[C@H]3CC[C@H](N)C3)nn2-c2ccc(C#N)cc2)on1	**390**
**14**	O=C(Nc1cccc2c1N(Cc1ccc(OC3CCNCC3)cc1)CCC2)c1cc2ccccc2n1CC1CC1	**442**
**15**	Cc1cc(COc2cc(C(=O)N3CC[C@H](F)[C@H](N)C3)nn2-c2ccc(C#N)cc2)on1	**950**

**16**	CN1CCC(Oc2ccc(OCc3cccc(NC(=O)c4cc5sccc5n4C)c3)cc2)CC1	**1400**
**17**	Cc1ccc([C@@H]2CN(C(=O)CC3CCNCC3)C[C@@H]2c2ccc(C#N)cc2)cc1	**2290**
**18**	O=C(Nc1cccc2c1N(Cc1ccc(OC3CCNCC3)cc1)CCC2)c1cc2ccccc2n1C(F)F	**2590**
**19**	CN1CCN(C(=S)SCC(=O)Nc2ccc(C(=O)/C=C/c3cccc(F)c3)cc2)CC1	**2600**
**20**	CCc1cn(C)c(-c2cc3sccc3n2C)n1	**2650**
**21**	CCN1CCN(C(=S)SCC(=O)Nc2ccc(C(=O)/C=C/c3cccc(F)c3)cc2)CC1	**6030**
**22**	O=C(/C=C/c1cccc(Br)c1)Nc1cccc(OCC2CCNCC2)c1	**19690**
**23**	N/C(=N\O)c1cccc(/C=C/c2cc(O)cc(O)c2)c1	**25700**
**24**	Cn1c(C(=O)Nc2ccccc2)cc2sccc21	**33900**

Table S3. The drug-likeness and ADMET properties of compounds screened after visualized analysis through SBVS.

**Ligand**	**Linpinski's+Veber's**							**ADMET**				
	**LogP[1]**	**MW[1]**	**nHA[1]**	**nHD[1]**	**TPSA[1]**	**nRot[1]**	**PAINS**	**Absorption_level[2]**	**Solubility_level[3]**	**BBB_level[4]**	**Ames[5]**	**WOE[5]**
**1**	1.24	407.35	5	0	71.97	4	0	0	3	3	0.16	0.25
**2**	2.66	477.61	4	1	78.95	10	0	0	3	3	0.06	0.27
**3**	1.59	353.42	4	2	98.65	7	0	0	3	3	0.24	0.27
**4**	0.27	354.37	4	1	90.78	4	0	0	3	3	0.01	0.27
**5**	2.74	321.38	2	3	73.99	7	0	0	3	3	0.20	0.30
**6**	2.78	354.38	3	1	60.85	4	0	0	3	2	0.15	0.26

1: LogP: Lipophilicity, MW: Molecular weight, nHA: Number of hydrogen bond acceptors, nHD: Number of hydrogen bond donors, TPSA: Topological polar surface area, nRot: Number of rotatable bonds

2: Absorption_levels: 0 (very good), 1 (good), 2 (low) and 3 (very low).

3: Solubility_level: 0 (extremely low), 1 (very low), 2 (low), 3 (good), 4(very good), 5 (very high) and 6 (unknown)

4: Blood–brain barrier (BBB) levels: 0 (very high penetrant), 1 (high), 2 (medium), 3 (low), 4 (undefined) and 5 (unknown)

5: Ames: Ames mutagenicity, WOE: Weight-of-evidence rodent and Carcinogenicity

Table S4. Calculated RMSD values of complexes in the MD simulations through SBVS and LBVS.

Complex	RMSD (Å) (SBVS)	Complex	RMSD (Å) (LBVS)
**LSD1**	3.45	**LSD1**	3.45
**LSD1_CC-90011**	3.25	**LSD1_CC-90011**	3.25
**LSD1_Lig1**	4.01	**LSD1_Com1**	2.91
**LSD1_Lig2**	2.78	**LSD1_Com2**	3.31
**LSD1_Lig3**	2.99	**LSD1_Com3**	3.42
**LSD1_Lig4**	2.59	**LSD1_Com4**	2.74
**LSD1_Lig5**	3.55	**LSD1_Com5**	3.23
**LSD1_Lig6**	3.35	**LSD1_Com6**	4.36
		**LSD1_Com7**	3.43

Table S5. Free energy decomposition calculations for the binding pocket-composed residues in **LSD1_CC-90011** and **LSD1_Lig2** complexes through SBVS.

Complexs	Residue Number	Van der Waals			Electrostatic			Polar Solvation			Total		
		Avg.	Std. Dev.	Std.Err. of Mean	Avg.	Std. Dev.	Std.Err. of Mean	Avg.	Std. Dev.	Std.Err. of Mean	Avg.	Std. Dev.	Std.Err. of Mean
**LSD1_ CC-90011**	GLY330	-0.21	0.05	0.001	1.34	0.26	0.004	-0.37	0.05	0.001	0.77	0.23	0.003
	MET332	-0.97	0.20	0.003	0.40	0.21	0.003	-0.01	0.07	0.001	-0.59	0.27	0.004
	VAL333	-1.33	0.27	0.004	0.00	0.12	0.002	0.02	0.04	0.001	-1.32	0.29	0.004
	THR335	-0.06	0.03	0.000	0.14	0.09	0.001	-0.04	0.02	0.000	0.04	0.06	0.001
	ILE356	-0.53	0.18	0.003	-0.13	0.12	0.002	0.04	0.02	0.000	-0.63	0.20	0.003
	GLN358	-0.03	0.03	0.000	0.03	0.13	0.002	-0.01	0.03	0.000	-0.01	0.11	0.002
	PHE538	-2.29	0.38	0.005	-1.10	0.31	0.004	0.68	0.14	0.002	-2.71	0.46	0.007
	ALA539	-2.57	0.39	0.005	-0.36	0.36	0.005	0.59	0.10	0.001	-2.34	0.46	0.006
	ASN540	-0.82	0.24	0.003	-0.48	0.35	0.005	0.28	0.13	0.002	-1.01	0.36	0.005
	TRP552	-0.10	0.06	0.001	0.00	0.21	0.003	0.01	0.08	0.001	-0.09	0.17	0.002
	ASP555	-0.15	0.24	0.003	-2.61	2.88	0.041	0.91	1.11	0.016	-1.86	1.94	0.027
	HIS564	-0.33	0.18	0.003	0.22	1.24	0.018	0.17	0.37	0.005	0.06	1.01	0.014
	LEU659	-0.63	0.22	0.003	0.05	0.08	0.001	0.02	0.02	0.000	-0.57	0.22	0.003
	LYS661	-0.22	0.32	0.004	-8.26	1.09	0.015	2.53	0.33	0.005	-5.95	0.86	0.012
	LEU677	-0.34	0.16	0.002	0.01	0.05	0.001	0.01	0.01	0.000	-0.33	0.16	0.002

	TRP695	-1.40	0.38	0.005	-0.13	0.28	0.004	0.25	0.07	0.001	-1.28	0.44	0.006
	LEU706	-0.41	0.13	0.002	-0.06	0.08	0.001	0.06	0.02	0.000	-0.42	0.13	0.002
	TYR761	-1.50	0.39	0.005	0.27	0.27	0.004	0.19	0.07	0.001	-1.04	0.33	0.005
	SER762	-0.09	0.03	0.000	-0.33	0.23	0.003	0.14	0.09	0.001	-0.29	0.18	0.003
	PRO808	-0.70	0.18	0.003	-0.71	0.83	0.012	0.31	0.17	0.002	-1.10	0.73	0.010
	ALA809	-0.71	0.19	0.003	0.05	0.15	0.002	0.05	0.09	0.001	-0.61	0.22	0.003
	THR810	-0.75	0.18	0.003	0.12	0.22	0.003	0.01	0.06	0.001	-0.63	0.24	0.003
	FAD	-2.11	0.31	0.004	-0.46	0.30	0.004	0.51	0.09	0.001	-2.06	0.42	0.006
**LSD1_ Lig2**	GLY330	-0.04	0.01	0.000	0.09	0.04	0.001	-0.03	0.02	0.000	0.02	0.04	0.001
	MET332	-1.44	0.27	0.004	-0.71	0.36	0.005	0.24	0.06	0.001	-1.90	0.44	0.006
	VAL333	-2.27	0.32	0.005	-0.28	0.21	0.003	0.07	0.08	0.001	-2.47	0.37	0.005
	THR335	-0.67	0.23	0.003	-0.26	0.60	0.009	0.11	0.11	0.002	-0.81	0.53	0.007
	ILE356	-0.72	0.21	0.003	-0.12	0.08	0.001	0.04	0.08	0.001	-0.80	0.22	0.003
	GLN358	-2.01	0.41	0.006	-0.29	0.31	0.004	0.26	0.12	0.002	-2.03	0.50	0.007
	PHE538	-1.74	0.35	0.005	-0.56	0.30	0.004	0.45	0.16	0.002	-1.85	0.52	0.007
	ALA539	-2.06	0.35	0.005	-0.70	0.36	0.005	0.63	0.11	0.002	-2.12	0.44	0.006
	ASN540	-1.57	0.29	0.004	-0.61	0.26	0.004	0.67	0.15	0.002	-1.52	0.43	0.006
	TRP552	-0.34	0.33	0.005	-0.18	0.18	0.003	0.17	0.16	0.002	-0.36	0.33	0.005
	ASP555	-0.02	0.01	0.000	-0.60	0.22	0.003	0.15	0.07	0.001	-0.47	0.16	0.002
	HIS564	-0.95	0.30	0.004	-0.76	0.58	0.008	0.63	0.26	0.004	-1.07	0.61	0.009

LEU659	-0.64	0.17	0.002	0.02	0.04	0.001	0.00	0.02	0.000	-0.63	0.17	0.002
LYS661	-0.06	0.01	0.000	-1.62	0.26	0.004	0.52	0.11	0.002	-1.17	0.26	0.004
LEU677	-0.58	0.22	0.003	-0.09	0.06	0.001	0.03	0.02	0.000	-0.63	0.24	0.003
TRP695	-0.33	0.09	0.001	-0.95	0.19	0.003	0.40	0.11	0.002	-0.88	0.21	0.003
LEU706	-0.18	0.06	0.001	-0.06	0.04	0.001	0.02	0.02	0.000	-0.22	0.07	0.001
TYR761	-2.29	0.32	0.005	-0.22	0.29	0.004	0.21	0.07	0.001	-2.29	0.48	0.007
SER762	-0.21	0.18	0.002	-0.83	0.19	0.003	0.41	0.06	0.001	-0.63	0.17	0.002
PRO808	-2.39	0.36	0.005	-0.48	0.22	0.003	0.39	0.11	0.002	-2.48	0.40	0.006
ALA809	-1.66	0.25	0.004	0.41	0.13	0.002	-0.03	0.05	0.001	-1.29	0.23	0.003
THR810	-1.06	0.29	0.004	-0.03	0.26	0.004	0.14	0.07	0.001	-0.96	0.42	0.006
FAD	-2.75	0.47	0.007	-1.73	0.52	0.007	0.63	0.10	0.001	-3.85	0.63	0.009

Table S6. Experimental and estimated activities of 24 compounds based on HypoGen1 in the test set.

**Compound**	**Experimental IC_{50} (nM)**	**Estimated IC_{50} (nM)**	**Error[1]**	**Experimental Scale[2]**	**Estimated Scale[2]**	**Fit Value[3]**
**1**	0.70	0.23	-3.10	+++	+++	9.77
**2**	0.80	0.21	-3.77	+++	+++	9.80
**3**	1.40	0.21	-6.75	+++	+++	9.81
**4**	4.90	16.09	3.28	+++	+++	7.92
**5**	9.20	15.56	1.69	+++	+++	7.93
**6**	18.00	161.62	8.98	+++	+++	6.91
**7**	57.00	17.34	-3.29	+++	+++	7.89
**8**	74.60	108.85	1.46	+++	+++	7.09
**9**	79.00	109.01	1.38	+++	+++	7.09
**10**	85.70	359.08	4.19	+++	++	6.57
**11**	121.00	16.18	-7.48	+++	+++	7.91
**12**	200.00	176.50	-1.13	+++	+++	6.88
**13**	390.00	2145.74	5.50	++	+	5.79
**14**	442.00	1264.82	2.86	++	+++	6.02
**15**	950.00	2009.67	2.12	++	+	5.82
**16**	1400.00	606.19	-2.31	++	++	6.34
**17**	2290.00	5831.73	2.55	+	+	5.36
**18**	2590.00	5914.44	2.28	+	+	5.35
**19**	2600.00	887.87	-2.93	+	++	6.17
**20**	2650.00	658.63	-4.02	+	++	6.30
**21**	6030.00	2710.09	-2.23	+	+	5.69
**22**	19690.00	5814.97	-3.39	+	+	5.36
**23**	25700.00	5975.70	-4.30	+	+	5.35
**24**	33900.00	5834.15	-5.81	+	+	5.36

1: Difference between the predicted and experimental activity values.

2: Most active (<200 nM, +++), moderately active (200-2000 nM, ++), and inactive (>2000 nM, +).

3: Fit value indicated how well the features in the pharmacophore overlap the chemical features in the molecule.

Table S7. The drug-likeness and ADMET properties of compounds screened after ADMET analysis through LBVS.

Compound	Linpinski's+Veber's						PAINS	ADMET									
	LogP[1]	MW[1]	nHA[1]	nHD[1]	TPSA[1]	nRot[1]		HIA[2]	Caco-2[2]	MDCK (10^{-6})[2]	BBB[2]	PPB[2]	hERG[3]	H-HT[3]	Ames[3]	ROA[3]
**1**	1.13	345.17	7	4	100.95	5	0	0.022	-5.03	8.97	0.07	86.39%	0.07	0.25	0.13	0.11
**2**	2.26	378.25	6	3	79.82	11	0	0.002	-4.66	47.80	0.39	75.36%	0.35	0.16	0.02	0.03
**3**	3.88	508.28	10	2	119.50	10	0	0.005	-4.84	2.28	0.45	89.76%	0.22	0.40	0.02	0.02
**4**	3.66	494.26	10	2	119.50	9	0	0.010	-4.86	25.00	0.46	88.71%	0.34	0.32	0.02	0.02
**5**	1.86	372.22	7	1	72.50	6	0	0.005	-5.06	15.10	0.45	51.69%	0.09	0.35	0.02	0.16
**6**	2.11	336.11	5	1	48.47	6	0	0.031	-4.69	80.10	0.19	63.98%	0.32	0.20	0.03	0.30
**7**	2.72	463.19	10	1	116.70	8	0	0.008	-4.71	26.30	0.23	83.08%	0.11	0.15	0.02	0.004

1: LogP: Lipophilicity, MW: Molecular weight, nHA: Number of hydrogen bond acceptors, nHD: Number of hydrogen bond donors, TPSA: Topological polar surface area, nRot: Number of rotatable bonds,

2: HIA: Human intestinal absorption, Caco-2: Caco-2 permeability, MDCK: Madin−darby canine kidney permeability, BBB: Blood–brain barrier, PPB: plasma protein binding hepatotoxicity; HIA values were set less than 0.5 to have good absorption properties. To have a proper permeability, Caco-2 and MDCK values were set higher than -5.15 log cm/s and 2×10^{-6} cm/s, respectively. BBB values were set less than 0.5 to block the blood-brain barrier and to avoid CNS side effects. PPB values were less than 90% in order to have an appropriate oral bioavailability.

3: hERG: the human ether-a-go-go related gene, H-HT: The human hepatotoxicity, Ames: Ames mutagenicity, ROA: Rat oral acute toxicity; The values of H-HT, hERG, AMES and ROA were set less than 0.5 to avoid liver injury, impairment of cardiac function, mutagenic and toxic.

Table S8. Free energy decomposition calculations for the binding pocket-composed residues in **LSD1_Comp2**, **LSD1_Comp3**, **LSD1_Comp4** and **LSD1_Comp7** complexes through LBVS.

	Residue Number	Van der Waals			Electrostatic			Polar Solvation			Total		
		Avg.	Std. Dev.	Std.Err. of Mean	Avg.	Std. Dev.	Std.Err. of Mean	Avg.	Std. Dev.	Std.Err. of Mean	Avg.	Std. Dev.	Std.Err. of Mean
**LSD1_Comp2**	GLY330	-0.01	0.00	0.000	-0.05	0.05	0.001	0.00	0.02	0.000	-0.06	0.05	0.001
	MET332	-0.09	0.03	0.000	-0.17	0.07	0.001	0.07	0.02	0.000	-0.20	0.06	0.001
	VAL333	-1.26	0.31	0.004	0.34	0.12	0.002	0.02	0.06	0.001	-0.90	0.33	0.005
	THR335	-0.82	0.28	0.004	0.14	0.06	0.001	-0.04	0.09	0.001	-0.71	0.29	0.004
	ILE356	-0.04	0.02	0.000	-0.01	0.02	0.000	0.01	0.01	0.000	-0.04	0.02	0.000
	GLN358	-0.01	0.01	0.000	0.01	0.02	0.000	0.00	0.01	0.000	0.00	0.02	0.000
	PHE538	-0.21	0.13	0.002	-0.08	0.09	0.001	0.01	0.04	0.001	-0.28	0.13	0.002
	ALA539	-0.88	0.32	0.005	-0.91	0.37	0.005	0.38	0.11	0.002	-1.41	0.49	0.007
	ASN540	-1.45	0.39	0.005	-1.04	0.68	0.010	0.85	0.13	0.002	-1.64	0.83	0.012
	TRP552	-0.91	0.29	0.004	-0.13	0.16	0.002	0.31	0.13	0.002	-0.74	0.29	0.004
	ASP555	-0.30	0.62	0.009	-5.60	3.73	0.053	2.37	1.42	0.020	-3.53	2.33	0.033
	HIS564	-0.96	0.27	0.004	0.90	0.44	0.006	0.19	0.19	0.003	0.13	0.44	0.006
	LYS661	-0.01	0.00	0.000	-0.85	0.27	0.004	0.33	0.12	0.002	-0.54	0.28	0.004
	LEU677	-0.01	0.01	0.000	0.00	0.01	0.000	0.00	0.00	0.000	-0.01	0.01	0.000
	TRP695	-0.03	0.02	0.000	0.03	0.02	0.000	0.00	0.01	0.000	-0.01	0.03	0.000

	LEU706	-0.02	0.01	0.000	-0.03	0.02	0.000	0.02	0.01	0.000	-0.03	0.02	0.000
	TYR761	-1.70	0.29	0.004	-0.03	0.55	0.008	0.10	0.15	0.002	-1.63	0.52	0.007
	SER762	-0.39	0.10	0.001	-0.29	0.19	0.003	0.53	0.13	0.002	-0.15	0.20	0.003
	PRO808	-1.86	0.38	0.005	0.53	0.37	0.005	0.29	0.16	0.002	-1.04	0.45	0.006
	ALA809	-1.29	0.26	0.004	0.11	0.17	0.002	0.10	0.10	0.001	-1.08	0.30	0.004
	THR810	-1.72	0.29	0.004	-0.73	0.23	0.003	0.15	0.07	0.001	-2.31	0.39	0.005
	FAD	-2.31	0.37	0.005	-1.52	0.43	0.006	0.48	0.11	0.002	-3.35	0.47	0.007
**LSD1_Comp3**	GLY330	-0.01	0.00	0.000	-0.05	0.05	0.001	0.00	0.02	0.000	-0.06	0.05	0.001
	MET332	-0.14	0.14	0.002	0.04	0.04	0.001	0.03	0.03	0.000	-0.08	0.14	0.002
	VAL333	-0.68	0.24	0.003	0.10	0.06	0.001	0.01	0.04	0.001	-0.57	0.23	0.003
	THR335	-0.31	0.23	0.003	0.08	0.09	0.001	0.00	0.04	0.001	-0.23	0.21	0.003
	ILE356	-0.01	0.01	0.000	-0.03	0.04	0.000	0.00	0.01	0.000	-0.05	0.04	0.001
	GLN358	-0.33	0.47	0.007	0.01	0.33	0.005	0.07	0.18	0.003	-0.25	0.46	0.007
	PHE538	-0.75	0.29	0.004	-0.08	0.13	0.002	0.14	0.11	0.002	-0.69	0.28	0.004
	ALA539	-2.79	0.49	0.007	-0.79	0.54	0.008	0.58	0.18	0.002	-3.00	0.65	0.009
	ASN540	-2.50	0.62	0.009	-3.41	1.09	0.015	1.45	0.21	0.003	-4.46	1.04	0.015
	TRP552	-1.67	0.51	0.007	0.02	0.28	0.004	0.20	0.16	0.002	-1.45	0.52	0.007
	ASP555	-0.17	0.34	0.005	-1.02	2.48	0.035	0.45	0.97	0.014	-0.74	1.82	0.026
	HIS564	-0.41	0.36	0.006	1.30	1.38	0.019	-0.05	0.42	0.006	0.84	1.12	0.016
	LYS661	-0.02	0.01	0.000	-0.07	0.34	0.005	0.18	0.11	0.002	0.09	0.31	0.004

	LEU677	-0.02	0.05	0.001	0.00	0.02	0.000	0.01	0.01	0.000	-0.02	0.05	0.001
	TRP695	-0.02	0.01	0.000	0.03	0.03	0.000	0.01	0.01	0.000	0.02	0.03	0.000
	LEU706	-0.03	0.02	0.000	0.00	0.03	0.000	0.01	0.01	0.000	-0.02	0.02	0.000
	TYR761	-1.48	0.58	0.008	0.31	0.29	0.004	0.04	0.09	0.001	-1.13	0.48	0.007
	SER762	-0.32	0.16	0.002	-0.58	0.31	0.004	0.45	0.20	0.003	-0.45	0.25	0.003
	PRO808	-0.34	0.14	0.002	-0.15	0.14	0.002	0.08	0.06	0.001	-0.41	0.19	0.003
	ALA809	-0.58	0.26	0.004	0.27	0.18	0.003	0.07	0.11	0.002	-0.25	0.27	0.004
	THR810	-0.24	0.13	0.002	0.02	0.08	0.001	0.01	0.03	0.000	-0.21	0.14	0.002
	FAD	-0.83	0.31	0.004	-0.31	0.17	0.002	0.36	0.19	0.003	-0.77	0.32	0.004
**LSD1_Comp4**	GLY330	-0.08	0.04	0.001	-0.17	0.07	0.001	-0.19	0.06	0.001	-0.43	0.11	0.002
	MET332	-1.79	0.38	0.005	0.07	0.16	0.002	0.14	0.09	0.001	-1.58	0.40	0.006
	VAL333	-2.16	0.56	0.008	-0.08	0.21	0.003	0.03	0.05	0.001	-2.21	0.64	0.009
	THR335	-0.16	0.18	0.003	-0.16	0.11	0.002	0.02	0.03	0.000	-0.30	0.20	0.003
	ILE356	-0.89	0.28	0.004	0.02	0.10	0.001	0.04	0.03	0.000	-0.83	0.31	0.004
	GLN358	-0.26	0.50	0.007	-0.04	0.71	0.010	0.07	0.22	0.003	-0.23	0.78	0.011
	PHE538	-2.51	0.44	0.006	-0.29	0.31	0.004	0.62	0.14	0.002	-2.18	0.56	0.008
	ALA539	-2.80	0.38	0.005	-0.18	0.37	0.005	0.46	0.15	0.002	-2.52	0.44	0.006
	ASN540	-0.83	0.44	0.006	-0.41	0.57	0.008	0.42	0.29	0.004	-0.82	0.61	0.009
	TRP552	-0.94	0.58	0.008	-0.06	0.07	0.001	0.25	0.17	0.002	-0.76	0.47	0.007
	ASP555	-0.89	0.42	0.006	0.56	0.70	0.010	0.21	0.24	0.003	-0.12	0.52	0.007

	HIS564	-0.41	0.23	0.003	-2.69	1.95	0.028	0.76	0.50	0.007	-2.34	1.52	0.021
	LYS661	-0.97	0.05	0.001	-1.18	0.35	0.005	0.11	0.30	0.004	-2.04	0.46	0.006
	LEU677	-0.57	0.21	0.003	0.03	0.08	0.001	0.03	0.02	0.000	-0.51	0.23	0.003
	TRP695	-0.81	0.29	0.004	0.04	0.08	0.001	0.21	0.09	0.001	-0.56	0.29	0.004
	LEU706	-0.39	0.15	0.002	0.09	0.05	0.001	0.09	0.03	0.000	-0.21	0.15	0.002
	TYR761	-1.46	0.58	0.008	0.03	0.41	0.006	0.29	0.09	0.001	-1.14	0.68	0.010
	SER762	-0.05	0.03	0.000	-0.05	0.08	0.001	0.05	0.03	0.000	-0.06	0.08	0.001
	PRO808	-0.31	0.20	0.003	0.25	0.38	0.005	0.04	0.08	0.001	-0.02	0.29	0.004
	ALA809	-0.44	0.24	0.003	0.15	0.25	0.003	0.13	0.15	0.002	-0.16	0.31	0.004
	THR810	-0.33	0.26	0.004	-0.13	0.17	0.002	0.05	0.05	0.001	-0.41	0.34	0.005
	FAD	-1.77	0.55	0.008	-0.95	0.56	0.008	0.64	0.16	0.002	-2.08	0.87	0.012
**LSD1_Comp7**	GLY330	-0.02	0.00	0.000	0.04	0.06	0.001	-0.05	0.03	0.000	-0.02	0.07	0.001
	MET332	-0.89	0.28	0.004	-0.65	0.34	0.005	0.29	0.09	0.001	-1.25	0.37	0.005
	VAL333	-1.27	0.31	0.004	-0.04	0.13	0.002	0.06	0.04	0.001	-1.25	0.29	0.004
	THR335	-0.07	0.03	0.000	0.08	0.04	0.001	-0.02	0.01	0.000	-0.01	0.04	0.001
	ILE356	-0.37	0.20	0.003	-0.05	0.06	0.001	0.03	0.04	0.000	-0.38	0.19	0.003
	GLN358	-0.14	0.19	0.003	0.02	0.17	0.002	0.02	0.10	0.001	-0.10	0.19	0.003
	PHE538	-1.29	0.29	0.004	-0.13	0.26	0.004	0.16	0.08	0.001	-1.26	0.35	0.005
	ALA539	-3.15	0.43	0.006	-1.06	0.47	0.007	0.64	0.09	0.001	-3.57	0.58	0.008
	ASN540	-1.41	0.39	0.005	-1.96	0.69	0.010	0.66	0.14	0.002	-2.70	0.61	0.009

TRP552	-1.47	0.57	0.008	-0.75	0.51	0.007	0.48	0.26	0.004	-1.75	0.74	0.010
ASP555	-0.02	0.02	0.000	-0.49	0.24	0.003	0.21	0.07	0.001	-0.30	0.19	0.003
HIS564	-1.40	0.45	0.006	-1.10	1.66	0.023	1.13	0.65	0.009	-1.37	1.47	0.021
LYS661	-0.02	0.01	0.000	-1.12	0.57	0.008	0.48	0.20	0.003	-0.67	0.52	0.007
LEU677	-0.22	0.14	0.002	-0.01	0.03	0.000	0.02	0.01	0.000	-0.21	0.13	0.002
TRP695	-0.06	0.03	0.000	-0.02	0.10	0.001	0.03	0.04	0.001	-0.06	0.07	0.001
LEU706	-0.07	0.03	0.000	-0.06	0.05	0.001	0.03	0.01	0.000	-0.10	0.05	0.001
TYR761	-1.39	0.28	0.004	0.38	0.37	0.005	0.00	0.07	0.001	-1.02	0.30	0.004
SER762	-0.08	0.03	0.000	0.07	0.13	0.002	0.08	0.07	0.001	0.07	0.12	0.002
PRO808	-0.17	0.07	0.001	-0.06	0.13	0.002	0.07	0.08	0.001	-0.16	0.11	0.002
ALA809	-0.56	0.16	0.002	0.52	0.29	0.004	0.12	0.12	0.002	0.08	0.32	0.005
THR810	-0.18	0.08	0.001	-0.06	0.07	0.001	0.02	0.03	0.000	-0.21	0.10	0.001
FAD	-0.77	0.17	0.002	-0.34	0.31	0.004	0.35	0.14	0.002	-0.76	0.28	0.004

Table S9. Hydrophobic interaction analysis in **LSD1_Comp2** and **LSD1_Comp4** complexes through LBVS.

Complex	Acceptor	Donor	Frac
**LSD1_Comp2**	TYR_761@CZ	Com2@C8	99.71%
	FAD@C12	Com2@C2	95.76%
	THR_810@CG2	Com2@C8	91.29%
	ALA_809@CB	Com2@C19	90.16%
**LSD1_Comp4**	PHE_538@CZ	Com4@C16	95.62%
	ALA_539@CB	Com4@C20	66.41%
	FAD@C15	Com4@C16	64.93%
	VAL_333@CB	Com4@C26	62.74%

Figure S1. (**A**) RMSD and (**B**) RMSF results during the molecular dynamics simulations of **sole-LSD1**, **LSD1_CC-90011**, **LSD1_Lig1**, **LSD1_Lig2**, **LSD1_Lig3**, **LSD1_Lig4**, **LSD1_Lig5** and **LSD1_Lig6** systems through SBVS.

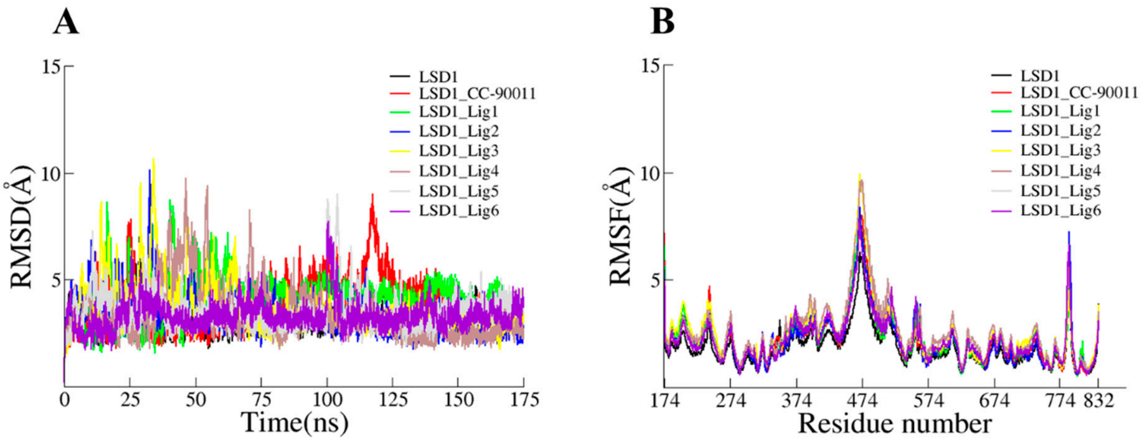

Figure S2. (**A**) RMSD and (**B**) RMSF results during the molecular dynamics simulations of **sole-LSD1**, **LSD1_CC-90011**, **LSD1_Comp1**, **LSD1_Comp2**, **LSD1_Comp3**, **LSD1_Comp4**, **LSD1_Comp5**, **LSD1_Comp6** and **LSD1_Comp7** systems through LBVS.

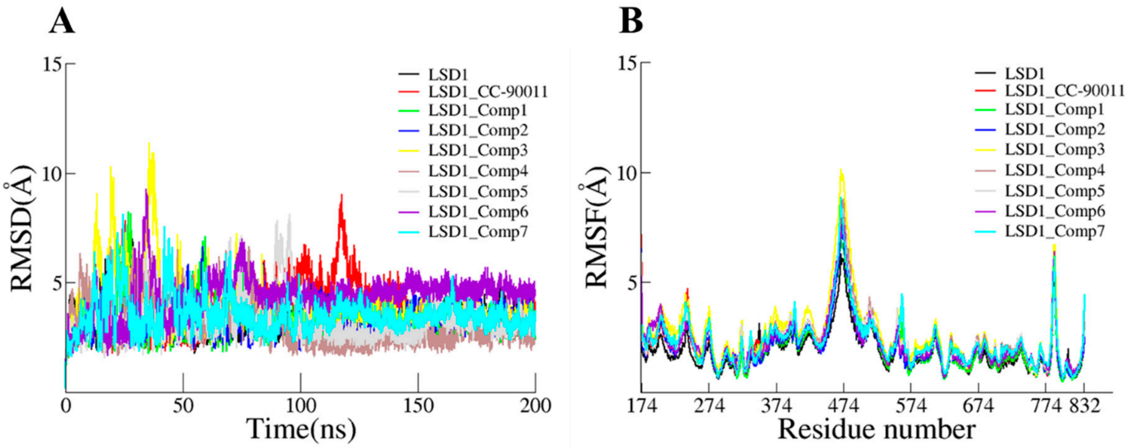

Supplement: Supplementary file 1 [file molecules-28-05315-s001.zip › molecules-2446779-supplementary.pdf]
